# The antennal scape organ of *Scutigera coleoptrata* (Myriapoda) and a new type of arthropod tip-pore sensilla integrating scolopidial components

**DOI:** 10.1186/s12983-021-00442-9

**Published:** 2021-11-04

**Authors:** Andy Sombke, Jörg Rosenberg, Gero Hilken, Carsten H. G. Müller

**Affiliations:** 1 Department of Evolutionary Biology, University of Vienna, Djerassiplatz 1, 1030 Vienna, Austria; 2 Soest, Germany; 3 Universitätsklinikum Essen, Zentrales Tierlaboratorium, Universität Duisburg-Essen, Hufelandstrasse 55, 45122 Essen, Germany; 4 Zoological Institute and Museum, University of Greifswald, Anklamer Strasse 20, 17489 Greifswald, Germany

**Keywords:** Antenna, Electron microscopy, Evolution, Mandibulata, Cone- and peg-shaped sensilla, Recto-canal epidermal glands, Scolopidium

## Abstract

**Background:**

Centipedes are terrestrial, predatory arthropods with specialized sensory organs. However, many aspects of their sensory biology are still unknown. This also concerns hygroreception, which is especially important for centipedes, as their epicuticle is thin and they lose water rapidly at low humidity. Thus, the detection of humid places is vital but to date no definite hygroreceptor was found in centipedes. House centipedes (Scutigeromorpha) possess a peculiar opening at the base of their antenna, termed ‘scape organ’, that houses up to 15 cone-shaped sensilla in a cavity. Lacking wall and tip-pores, these socket-less sensilla may be hypothesized to function as hygroreceptors similar to those found in hexapods.

**Results:**

The cone-shaped sensilla in the scape organ as well as nearby peg-shaped sensilla are composed of three biciliated receptor cells and three sheath cells. A tip-pore is present but plugged by a highly electron-dense secretion, which also overlays the entire inner surface of the cavity. Several solitary recto-canal epidermal glands produce the secretion. Receptor cell type 1 (two cells in cone-shaped sensilla, one cell in peg-shaped sensilla) possesses two long dendritic outer segments that project to the terminal pore. Receptor cell type 2 (one cell in both sensilla) possesses two shorter dendritic outer segments connected to the first (proximal) sheath cell that establishes a scolopale-like structure, documented for the first time in detail in a myriapod sensillum.

**Conclusions:**

The nearly identical configuration of receptor cells 1 with their long dendritic outer segments in both sensilla is similar to hexapod hygroreceptors. In *Scutigera coleoptrata*, however, the mechanism of stimulus transduction is different. Water vapor may lead to swelling and subsequent elongation of the plug pin that enters the terminal pore, thus causing stimulation of the elongated dendritic outer segments. The interconnection of receptor cell 2 with short outer dendritic segments to a scolopale-like structure potentially suits both sensilla for vibration or strain detection. Thus, both sensilla located at the antennal base of scutigeromorph centipedes fulfill a dual function.

**Supplementary Information:**

The online version contains supplementary material available at 10.1186/s12983-021-00442-9.

## Background

**Fig. 1:**
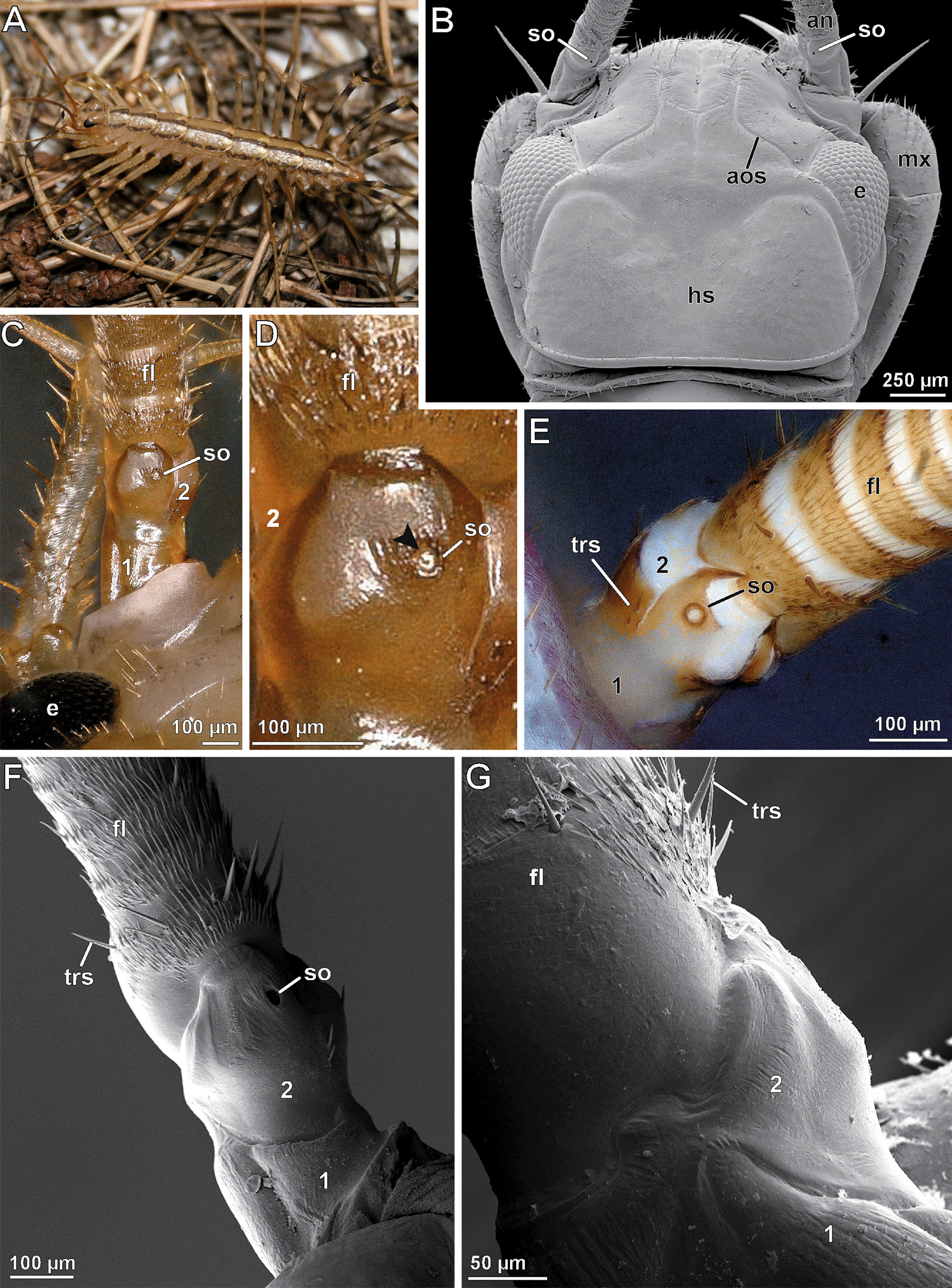
External morphology of the basal part of the antenna exhibiting the scape organ of *Scutigera coleoptrata*. **A** Habitus of *S. coleoptrata* (modified after [86]). **B** Head and basal antennal region of *S. coleoptrata* in dorsal view. Courtesy by Hans Pohl. **C** Dorsal face of antennal base in living state showing the fused scape and adjacent multi-annulated flagellum. The scape organ is present on the bulged area. **D** Scape organ in detailed view, magnified from C. Note that the pore is plugged by a white mass (arrowhead). **E** Dorsal face of antennal base in ethanol-fixed state. Note the coloration correlated to hardness degrees of the cuticle. **F** Dorsal face of the antennal base (left antenna, lateral side of the animal is to the left) comprising the two basal antennomeres and adjacent flagellomeres. The second (distal) antennomere bears the scape organ. **G** Ventrolateral face of the antennal base (left antenna). None of the antennomeres display any obvious cuticular microsculptures, trichomes or sensilla. an, antenna; aos, antero-ommatidial suture; e, compound eye; fl, flagellum; hs, head shield; mx, maxillipede (forcipule); so, scape organ; trs, trichoid sensillum; 1, first (proximal) antennomere; 2s (distal) antennomere (fused scape)

Centipedes are terrestrial, predatory arthropods with specialized sensory organs and a wide range of behavioral adaptations to detect and capture prey on and in the soil. As their epicuticle is thin and a wax layer is missing, they lose water rapidly at low humidity [1–3]. Thus, the detection of humid places is vital, but to date no definite hygroreceptor was found and described in detail in centipedes [4, 5]. Generally, they are widely understudied, especially on the ultrastructural level [6, 7]. Although progress was achieved in the past 20 years with respect to sensory organs (summarized in [4, 8]), there are still remarkable gaps of knowledge. One in particular is the ‘scape organ’ (also termed ‘shaft organ’) of scutigeromorph centipedes [9–11] (Fig. 1). In all representatives of Scutigeromorpha, the antennal scape (fusion of the two proximal antennomeres) houses the scape organ, which is a single, round opening with a diameter of 10 to 15 µm [4, 9–20]. In addition to the scape organ, the antennae of Scutigeromorpha are unique due to their enormous length as they are composed of up to 500 antennomeres including nodes enabling bending [11, 21]. Very little is known about the cellular organization and function of the scape organ. The only accounts available date back over a hundred years and are restricted to a short report by Verhoeff [13] and a brief histological description by Fuhrmann [10] (reproduced in Additional file 1) that came along with line drawings of limited resolution. Fuhrmann [10] depicted from longitudinal sections piles of what he identified as receptor cells, nested in a spacious sensory epithelium having a connection to the sensory cones within the scape organ (Additional file 1D). It was the latter line drawing, in particular, that was reproduced in reviews on centipede sensory anatomy (e.g. [1, 4, 22]). Further insights were provided from comprehensive SEM studies on morphology and distribution of antennal sensilla in *Scutigera coleoptrata* [11]. Though excluding anatomy, this study revealed the compact nature of the sensory cones that are inserted tightly at the bottom of a spacious cavity and appear to be covered by a liquid/mucoid film. No tip-pores were detected at the sensory cones ([11]; their Fig. 1C). The lack of ultrastructural, behavioral, and functional data makes it virtually impossible to go beyond speculations about the biological role of the scape organ. Fuhrmann [10] considered that it most probably detects olfactory cues. Sombke et al. [11] assumed a morphological correspondence with sensilla brachyconica or so-called large sensory cones present on the nodes of the antennae. Because of the protected position of the sensory cones within the cavity, only a contact-chemoreceptive or mechanoreceptive function was excluded [11].

The present study sets out to provide a first thorough morphological analysis of the scape organ of *S. coleoptrata* using multimodal microscopic techniques. Based on the extensive literature on hexapod sensilla, it is known that some function-related ultrastructures may indicate mechanisms of stimulus transduction and, therefore, allow for cautious assessments of receptor modality and function. Classic examples for such essential ultrastructures are tubular bodies indicating mechanoreception, or elongated cilia in combination with a tip-pore enabling gustation. Furthermore, wall pores, pore tubules, and/or multiple dendrite branchings within the shaft lumen may indicate olfaction (e.g. [23–26]). Poreless sensory cones or pegs belong to the class of so called no-pore sensilla with inflexible sockets, usually referred to as *’np*-sensilla’ [23] or ‘*np-is*-sensilla’ [27]. In hexapods and myriapods, these sensilla may show a basiconic, styloconic, or coeloconic appearance (e.g. [4, 8, 27, 28]). Interestingly, some of these antennal *np*-sensilla turned out to be bi- or trifunctional as they integrate thermo- and hygroreceptors and sometimes even scolopidia-like components of unknown function (e.g. [23, 27–32]). Since hygro- and thermoreceptors have not been clearly identified or located in centipedes, we evaluate if the cone-shaped sensilla in the scape organ may cover these functions. This evaluation also includes so far unnoticed peg-shaped sensilla located at either side of the scape organ.

## Results

### External morphology and histology of the scape

**Fig. 2:**
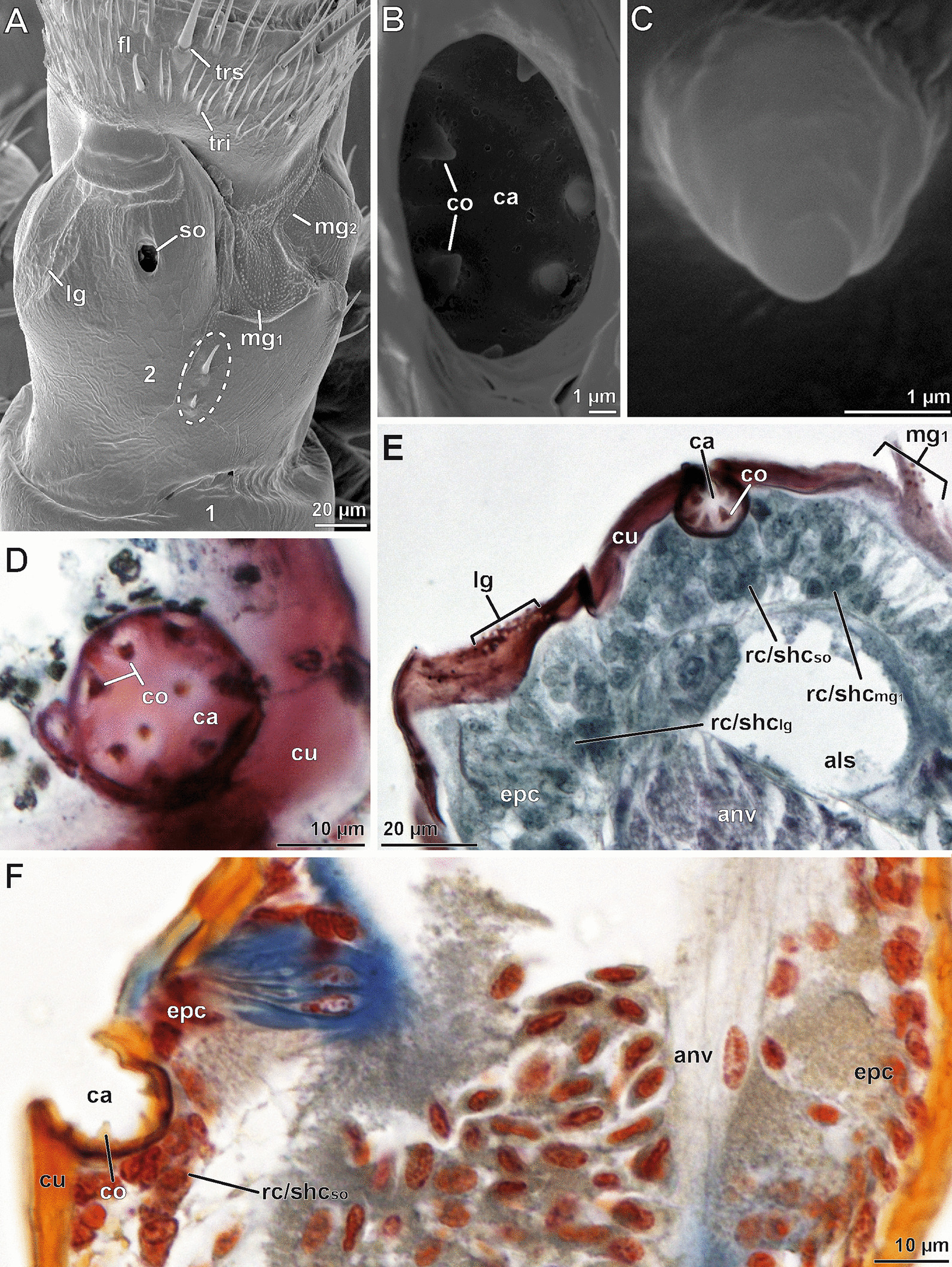
External morphology and histological anatomy of the scape organ and surrounding areas of the antennal base. **A** Dorsal face of the second (distal) antennomere showing the pore of the scape organ. Note the lateral and median groups of peg-shaped sensilla aligned in sickle- or chevron-like formations. Dashed area marks the two trichoid sensilla posteromedial of the scape organ. **B** Magnification of the pore documenting the bottom of the cavity along with six cone-shaped sensilla. **C** Close-up of a cone-shaped sensillum; a terminal pore is not noticeable. **D** Paraffin cross-section of the cavity illustrating nine cone-shaped sensilla located at the bottom and lower lateral parts. Bodian staining. **E** Transverse paraffin section through the dorsal face of the scape, cut at the level of the scape organ. The scape organ is underlain by a thickened epidermis comprising clusters of elongated, bottle-shaped and tightly adjoined epithelial cells. Note the lateral and median groups of peg-shaped sensilla and associated clusters of receptor and sheath cells. The antennal nerve exhibits a slight purple staining due to the effect of silver impregnation targeting neurofibrils. Bodian staining. **F** Longitudinal paraffin section of the scape. Note the wider opening of the cavity (as compared to **E**) caused by its ovoid shape and longitudinal orientation of the section. Azan staining. als, antennal lymph space; anv, antennal nerve; ca, cavity; co, sensory cone (of cone-shaped sensillum); cu, cuticle; epc, interstitial epidermal cells; fl ,flagellum; lg, lateral group of peg-shaped sensilla; mg_1_, medial group 1 of peg-shaped sensilla; mg_2_, medial group 2 of peg-shaped sensilla; rc/shc_lg_, cluster of receptor and sheath cells of lateral group of peg-shaped sensilla; rc/shc_mg1_, cluster of receptor and sheath cells of first medial group of peg-shaped sensilla; rc/shc_so_, cluster of receptor and sheath cells of the scape organ; so, scape organ; tri, trichome; trs, trichoid sensillum; 1, first (proximal) antennomere; 2s (distal) antennomere

**Fig. 3:**
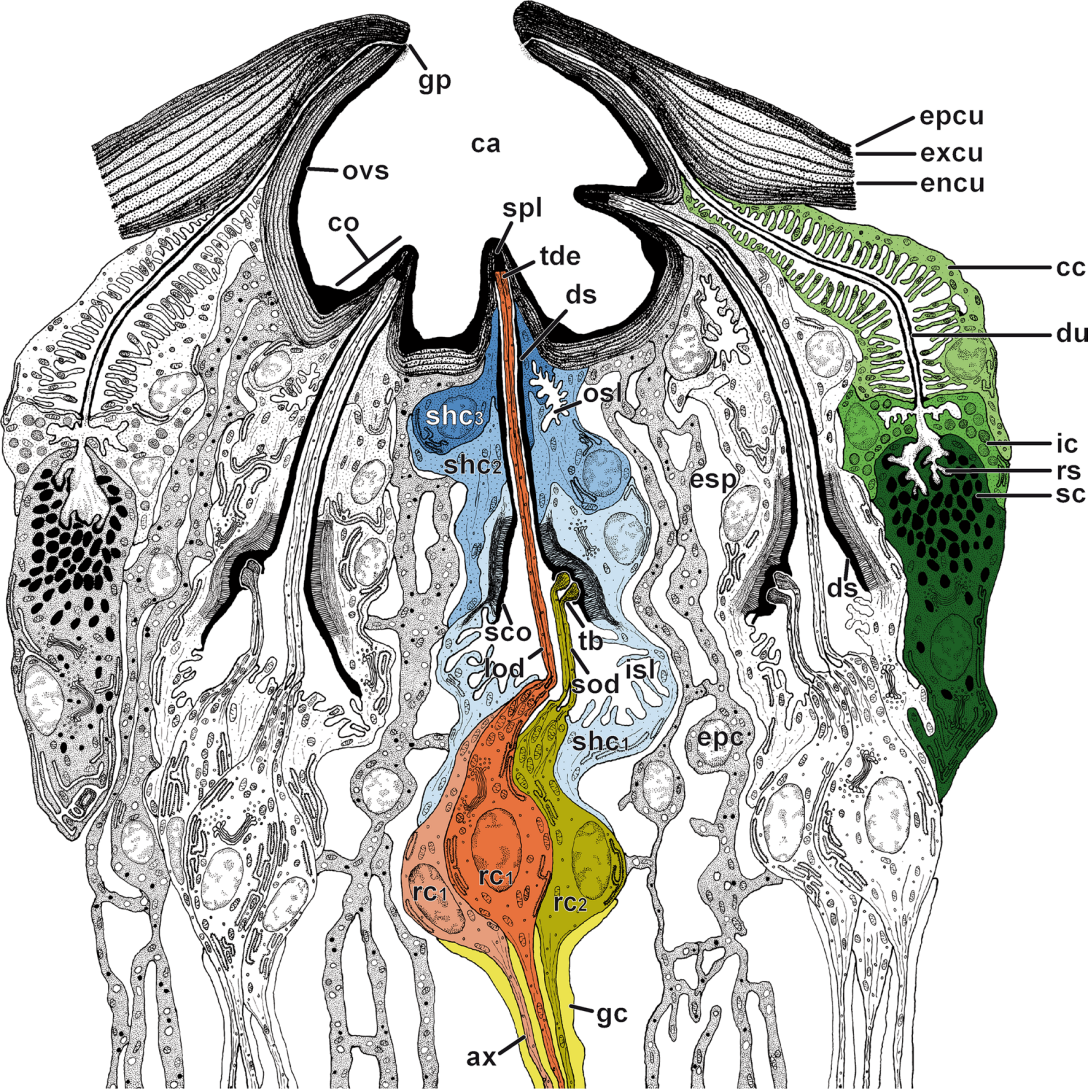
Cellular organization of scape organ of *Scutigera coleoptrata* shown in a semi-schematic reconstruction based on TEM analysis. Three cone-shaped sensilla and two recto-canal epidermal glands associated with the scape organ are cut longitudinally. Examples of cell types constituting the scape organ are color-coded in a single cone-shaped sensillum (at center; receptor cells stained in red, beige, and olive-green; glial cells in yellow; sheath cells in various shades of blue) and one gland (to the right; cells stained in various shades of green). The helical bending of the dendritic outer segments found below the sensory cones has not been reproduced in this 2D-reconstruction to keep the drawing simple and comprehensible. ax, receptor cell axon; cc, canal cell; ca, cavity; co, sensory cone (of cone-shaped sensillum); ds, dendritic sheath; du, conducting canal; encu, endocuticle; epc, interstitial epidermal cell; epcu, epicuticle; esp, extracellular space; excu, exocuticle; gc, glial cell; gp, glandular pore; ic, intermediary cell; isl, inner sensillum lymph space; lod, long dendritic outer segments; osl, outer sensillum lymph space; ovs, secretion layer; rc_1_, receptor cell with long dendritic outer segments; rc_2_, receptor cell with short dendritic outer segments; rs, reservoir; sc, secretory cell; sod, short dendritic outer segments; sco, scolopale-like structure; shc_1_, first (proximal) sheath cell (scolopale cell); shc_2_, second (median) sheath cell; shc_3_, third (distal) sheath cell; spl, secretion plug; tb, tubular bodies; tde, terminal structures of long dendrites (including tubular bodies)

**Fig. 4:**
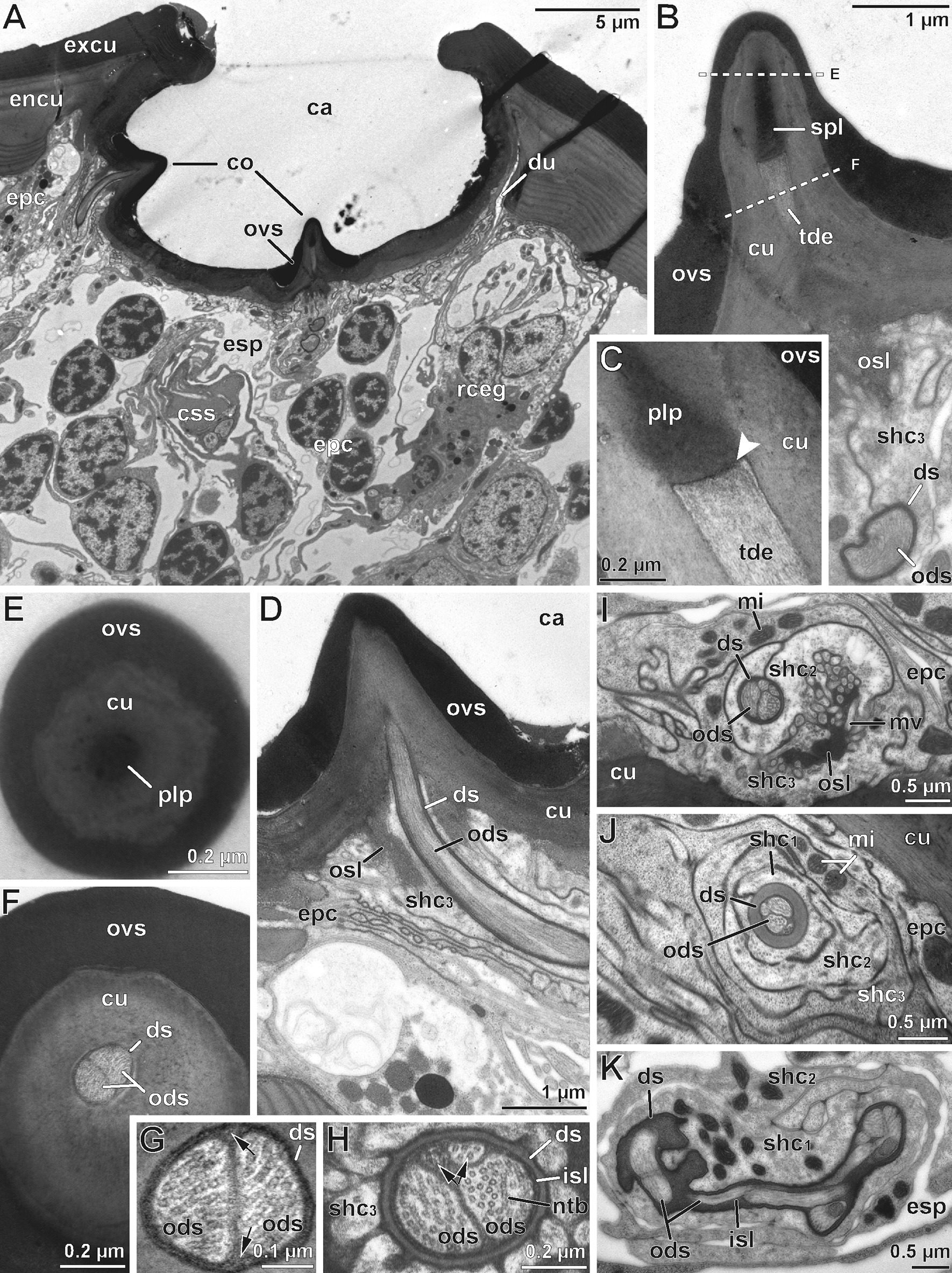
Internal ultrastructure of the scape organ based on TEM analysis: Overview and details of distal components with emphasis on the sensory apparatus. **A** Medio-longitudinal section of the sensory cavity with two cone-shaped sensilla, several clusters of receptor and sheath cells, epidermal cells, as well as of one associated recto-canal epidermal gland. **B** Detail of a cone-shaped sensillum in longitudinal section. Note the axial projection of the secretion layer plugging and concealing the terminal pore. **C** The lower tip of the secretion plug tightly adjoins the truncated distal tip of a long dendritic outer segment resembling a tubular body (arrowhead). **D** Para-longitudinal section of a cone-shaped sensillum showing some long outer dendritic segments, encapsulated by the dendritic sheath. **E–F** Cross-sections of a cone-shaped sensillum at a distal level through the secretion plug (**E**) and at a more proximal level through the tubular body-like terminations of two long outer dendritic segments (**F**). Section levels are indicated in (**B**). **G** Close-up of two long outer dendritic segments slightly below the section level shown in (**E**). Two small additional outer dendritic segments are visible between the two main ones (arrows), tubular body characteristics are not clearly discernible. **H** Cross-section approximately 5 µm below (**G**). Note the two smaller outer dendritic segments (arrows). **I–K** Sequence of consecutive cross-sections illustrating the sheath cell system and dendritic apparatus of receptor cells with long outer dendritic segments. **I** Immediately below the sensory cone. Note the presence of the minute outer sensillum lymph space. **J** Section level approximately 5 µm below (**I**), showing the transition zone where all three sheath cells overlap. Note also the increased width of the dendritic sheath. **K** Section level approximately 5 µm below (**J**), showing the coiled path of the ciliary apparatus. ca, cavity; co, sensory cone (of cone-shaped sensillum); css, cellular profile of cone-shaped sensillum; cu, cuticle; ds, dendritic sheath; du, conducting canal; encu, endocuticle; epc, interstitial epidermal cell; esp, extracellular space; excu, exocuticle; isl, inner sensillum lymph space; mi, mitochondria; mv, microvilli; ntb, neurotubules; ods, outer dendritic segment(s); osl, outer sensillum lymph space; ovs, secretion layer; rceg, recto-canal epidermal gland; css, cellular profile of cone-shaped sensillum; shc_1_, first (proximal) sheath cell (scolopale cell); shc_2_, second (median) sheath cell; shc_3_, third (distal) sheath cell; spl, secretion plug; tde, terminal structures of long outer dendritic segments

The scape organ of *Scutigera coleoptrata* is visible as a single opening, positioned at the dorsomedial side of the second antennomere (Figs. 1B–F, 2A; Additional file 1A). The location is characterized by a widely extended, moderately bulged surface. A proximal, stronger sclerotized region (dark brown coloration in living and fixed state) is separated from a distal, softer and light brown (white in fixed specimens) region by a sharp crest (compare Figs. 1C–G and 2A). The cuticular surface of the softer distal area displays numerous small peg-shaped sensilla, encompassed by a tuberculate cuticle that coincides with white, presumably softer cuticular areas in ethanol fixed specimens (compare Fig. 1E). In contrast, more lateral and ventral areas of the scape lack tuberculate areas and sensilla (Fig. 1F, G). The dorsolateral side of the scape is bulged (Fig. 2A). The lateral margin of the scape organ (adjoined by soft, tuberculate cuticle) bears a group of peg-shaped sensilla (Fig. 2A). Here, the crest projects coaxially to the antenna and thus separates these stronger sclerotized areas from the area housing the scape organ (Fig. 1C–F). At the dorsal side of the second antennomere, two strong trichoid sensilla are present (Figs. 1F, 2A), used here as an indication to distinguish between left and right antenna as well as dorsomedian (inner) and dorsolateral (outer) side. These features validate the drawing of Fuhrmann [10] (compare Additional file 1A). The spherical or ovoid pore opening of the scape organ is between 10 and 15 µm in diameter (Figs. 2A, B, 3, 4A), depending on size and age of the animal. In live animals, the pore (as well as the cavity underneath) is plugged by a white mass (Fig. 1C, D). Depending on pore dimensions and viewing angle, at least 4–7 pore-less sensory cones (2–3 µm in height) with rounded tips are visible inside the cavity (Figs. 2A, B, D–F; Additional file 1B). The surface of the cavity and the cones is smooth, occasionally displaying flat holes or thin cracks (Fig. 2B). Bodian-stained paraffin sections reveal the occurrence of usually 8–10 sensory cones in smaller adults (e.g. Fig. 2D, E), whereas larger individuals may possess up to 15 sensory cones. A distinctive sensory epithelium is present below the cavity (Fig. 2E, F). It contains piles of voluminous, bottle-shaped cells that are connected to the base of the sensory cones via more intensively stained, thread-like structures. The histological appearance of the sensory epithelium is inconsistent. In one specimen investigated, regular epidermal cells as well as receptor cells, sheath cells, and glandular cells constituting this epithelium are closely adjoined (Fig. 2E), while in two other specimens voluminous intercellular spaces are present (Fig. 2F). The sensory epithelium of the scape organ and neighboring groups of peg-shaped sensilla are close to the usually centered antennal nerve (Fig. 2E, F), which is the final target structure of primary afferents.

### Fine structural anatomy of cone-shaped sensilla of the scape organ

**Fig. 5:**
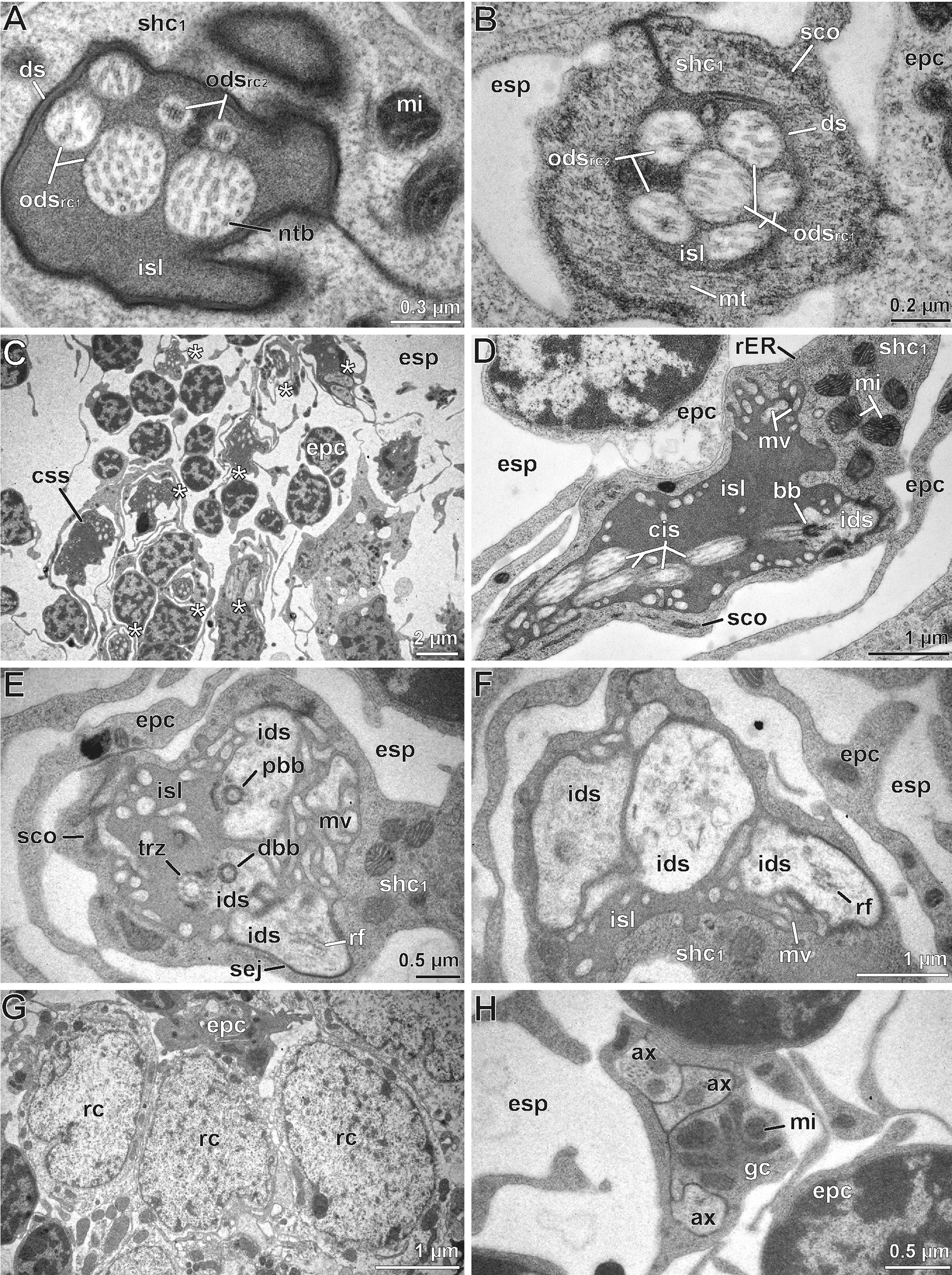
Internal ultrastructure of the scape organ based on TEM analysis: Overview and details of median and proximal components with focus on receptor cells with short outer dendritic segments and scolopidial elements. **A** Cross-section of inner sensillum lymph space housing the four long plus two short outer dendritic segments. The short outer dendritic segments are cut at a level where they just transform into tubular bodies. **B** Cross-section slightly proximal of (**A**), illustrating all six outer dendritic segments with similar diameters. Note the scolopale-like structure in the first sheath cell. **C** Slightly oblique overview of the median region of the receptor and accompanying sheath cell cluster (asterisks). Flattened, strongly stretched epidermal cells are cut at their nuclear level in between and outside receptor and sheath cell clusters, spread by an enormously expanded extracellular space. **D** Insertion zone of outer dendritic segments showing ciliary structures. Oblique-transverse section of the widened inner sensillum lymph space containing five profiles of outer dendritic segments. A further profile is cut at the transition zone of the outer and inner dendritic segment coinciding with the presence of a basal body. **E** Approximately the same region as shown in (**D**), but from a transverse perspective. All three inner dendritic segments are present. Two of them are cut at various levels of the basal body region. The mid profile displays two basal bodies underlining the biciliated nature of receptor cells. **F–H** Three cross sections taken from the proximal region of the receptor cell apparatus cut at the level of (**F**) dendritic inner segments, (**G**) the somata level, and (**H**) the axonic level. ax, receptor cell axon; bb, basal body/bodies; css, cellular profile of cone-shaped sensillum; dbb, distal basal body; ds, dendritic sheath; epc, interstitial epidermal cell; esp, extracellular space; gc, glial cell; ids, inner dendritic segment; isl, inner sensillum lymph space; mi, mitochondria; mv, microvilli; ntb, neurotubules; ods, outer dendritic segment(s); pbb, proximal basal body; rc, receptor cell; rER, rough endoplasmic reticulum; rf, ciliary root fibers; sco, scolopale-like structure; sej, septate junction; shc_1_, first sheath cell (scolopale cell); trz, transition zone (of the basal body)

**Fig. 6:**
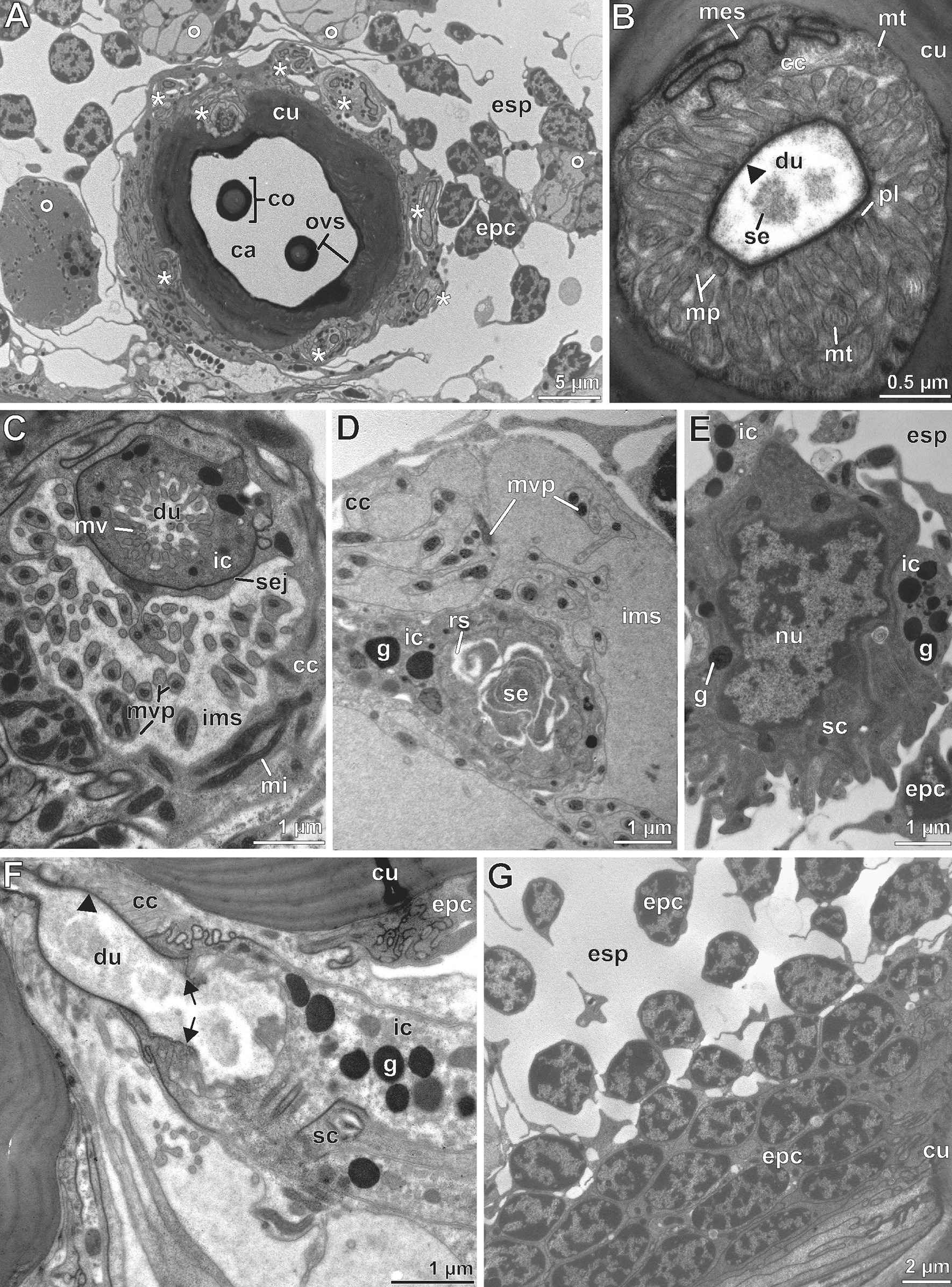
Internal ultrastructure of recto-canal epidermal glands associated with the scape organ based on TEM analysis. **A** Transverse section of the cavity with cross-profiles of two central cone-shaped sensilla and cellular profiles of eight further cone-shaped sensilla inserting higher in the cavity (asterisks). In addition, four associated epidermal glands (circles) are visible surrounding the sensory cavity. **B–E** Sequence of cross and cross-oblique sections illustrating the cellular organization of associated epidermal glands from the distal to proximal level. **B** Distal end of the canal cell piercing the cuticle. The widened conducting canal is covered by a distinct cuticle (arrowhead). **C** Cross section slightly below the transition zone of the canal cell and the intermediary cell. Note the non-cuticularized border of the conducting canal cut at the level of the intermediary cell. **D** Cross section of the apical aspect of the intermediary cell more proximal of (**C**). Note the apex, which is widely devoid of microvilli. The reservoir is clearly filled with secretion. **E** Lower third of the secretory cell accommodating the nucleus. The secretory cell is lined by densely granulated proximal processes of the intermediary cell. **F** Oblique-longitudinal section showing the canal and intermediary cells. The secretory cell is cut only tangentially. Note that the cuticular lining of the conducting canal starts at the distal part of the intermediary cells (arrows). Its proximal part is free of a cuticle. The duct-lining cuticle is marked by an arrowhead. **G** Oblique-horizontal section of epidermal cells forming the dome-shaped cuticular bulge of the scape organ. The epidermal cells are cubical and tightly adjoined to the periphery of the scape organ, but tend to become more distant towards its center. ca, cavity; cc, canal cell; co, sensory cone (of cone-shaped sensillum); cu, cuticle; du, conducting canal; esp, extracellular space; epc, interstitial epidermal cell; g, electron-dense granule; ic, intermediary cell; ims, intermicrovillar space of the canal cell; mes, mesaxonal membrane; mi, mitochondria; mv, microvilli; mvp, microvilliform process(es); mt, microtubules; mv, microvilli; nu, nucleus; ovs, secretion layer; pl, osmiophilic plaque (on microvillar tips); rs, reservoir; sc, secretory cell; se, secretion; sej, septate junction

TEM analysis revealed a remarkably voluminous and complex intercellular space reaching between the epithelial cells associated with sensory cones and associated epidermal glands, as well as between the regular (interstitial) epidermal cells. Thus, the entire sensory epithelium appears spongy and hardly coherent (Figs. 3, 4A, 5C). Interstitial epidermal cells are flattened and strongly ramified. Their nuclei are usually located far below the cuticle (Figs. 3, 4A, 5C, H, 6A). In the periphery of the scape organ, epidermal cells are compact and more regular in shape (Fig. 6G).

#### Receptor cells and ciliary apparatus

The sensory cones within the cavity are sensilla, henceforth termed as ‘cone-shaped sensilla’. They are innervated by three biciliated receptor cells (Fig. 3). Two of these receptor cells (termed rc1) possess long outer dendritic segments (= sensory cilia) that project into the cone (Figs. 3, 4A, B, D, F), where they terminate in a swollen structure containing numerous microtubules aligned in highly osmiophilic fibrillous material, strongly resembling tubular bodies (Fig. 4B, C, F, G). Two of these four long outer dendritic segments are larger and clearly reach the cone’s tip, whereas the other two are considerably thinner and hardly visible immediately below the cone’s tip (Fig. 4F, G, H). Distally, the tubular bodies of the two larger outer dendritic segments are truncated and directly adjoin an amorphous, extremely osmiophilic plug pin, which is continuous with the likewise extremely osmiophilic overlay lining the entire cuticle of the cavity (Figs. 3, 4B, C). This overlay most likely represents secretion produced by several recto-canal epidermal glands surrounding the scape organ (Fig. 3). Therefore, it is evident that the sensory cones bear a concealed terminal pore, which cannot be recognized by scanning electron microscopy.

The bundle of all outer dendritic segments projects through an extended extracellular space—the inner sensillum lymph space. Its distal and median tubular compartments are narrow and encompassed by a mostly thin, highly osmiophilic extracellular mantle—the dendritic sheath (Figs. 3, 4D, G, H, K, 5A, B). The proximal compartment of the inner sensillum lymph space is bulged and devoid of a dendritic sheath. Immediately below the sensory cone, the dendritic outer segments either project in a straight, upright course (as observed in cones located at the bottom of the sensory cavity; Fig. 3) or are slightly bent (as observed in cones located at the sidewall of the cavity; Figs. 3, 4D). In the distal area of the cone-shaped sensillum, the dendritic sheath is thickened over a short distance, where often all three sheath cells are present (Fig. 4J). Further proximally, the dendritic outer segments appear coiled in several loops (Fig. 4K). At the median level of the sensory cone, the dendritic outer segments resume their upright course. The inner sensillum lymph space continuously gets more expanded while the dendritic sheath withers (Figs. 3, 5A, B, D, E).

The median region of the cone-shaped sensillum is characterized by three features: (1) the presence of a third receptor cell (termed rc2) projecting a pair of short outer dendritic segments, (2) a transition zone of inner and outer dendritic segments (ciliary region), and (3) the presence of a scolopale-like structure (see discussion) produced by the first (proximal) sheath cell (Fig. 3). The shorter outer dendritic segments of the third receptor cell are recognizable by their terminal structures, which are attached to groove-like depressions inside the dendritic sheath. Like the terminations of the elongated outer dendritic segments, they exhibit characteristics of tubular bodies (microtubules encased in a highly electron-dense fibrillary matrix). However, they remain distinctly smaller than those of the first receptor cell(s) abutting the plug pin (0.1 *vs.* 0.2–0.3 µm in diameter). Within the ciliary region and immediately above the basal body, the microtubules of the three pairs of dendritic outer segments display a strictly ordered formation (Fig. 5D), which is a typical feature of primary (sensory) cilia. A few microns further proximally, each pair of these ciliary structures turns from the center to the periphery of the inner sensillum lymph space (Fig. 5D). The insertion of the ciliary structure at the tip of each inner dendritic segment is provided by two distinct basal body complexes (Fig. 5E), each comprising a typical top down sequence of the distal basal body (= distal centriole), the proximal basal body (= proximal centriole), and bundles of root fibers (according to [33]). The three inner dendritic segments extend further proximally, where they pass into a pile of receptor cell somata each including a large, polymorphic nucleus, which contains only small amounts of heterochromatin (Figs. 3, 5G). Proximally, each receptor cell soma tapers into a minute axonal process. Axons of each cone-shaped sensillum stay bundled and are encompassed by a sheath of glial cells (Figs. 3, 5H). Besides the cytoplasmic organelles mentioned above, receptor cells accommodate numerous, often elongated mitochondria of the cristae type. Further organelles commonly found in the soma are Golgi stacks, electron-dense spherules, and cisternae of the rough ER (e.g. Figs. 3, 5G). Bundles of receptor cell axons leave the sensory epithelium and join the antennal nerve.

#### Sheath cells

Each cone-shaped sensillum is associated with three different sheath cells that are classified according to their sequential appearance from proximal to distal parts of the sensory system, termed first (proximal), second (median), and third (distal) sheath cell. All three sheath cells are coiled around themselves at the apex, indicated by the presence of a mesaxonal membrane (Fig. 4I–K) and tend to merge in a telescope-like fashion towards the sensory cone (Figs. 3, 4J). The first sheath cell is the most voluminous and extended one. It tightly encompasses the most distal parts of the three receptor cell somata including the bundle of inner dendritic segments up to the region of the basal bodies (Figs. 3, 5D, F). Further distally, the first sheath cell surrounds the proximally widened compartment of the inner sensillum lymph space, which is invaded by numerous microvilli (Figs. 3, 5D–F). The apex of the first sheath cell abuts the dendritic sheath that encases the inner sensillum lymph space (Figs. 3, 4 J, K, 5A, B). The most characteristic feature of the first sheath cell is a complex cytoplasmic network of fibers and microtubules agglutinated in an extremely osmiophilic matrix (Figs. 3, 5B, D). Cross-sections reveal that this network establishes a circle at the periphery of the cell. Microtubules oriented perpendicular to this circular mass make contact with the outer face of the dendritic sheath (Figs. 3, 5B). At the base of the cell, the circular mass disintegrates by splitting into interconnected rods projecting towards various directions (Figs. 3, 5D–F). According to its ultrastructural appearance, this cytoplasmic network strongly resembles a scolopale, and therefore this sheath cell type is addressed here as scolopale-like cell. Besides scolopale-associated organelles, the first sheath cell is characterized by numerous mitochondria of the cristae type as well as dispersed cisternae of the rough and smooth ER, Golgi stacks, vesicles of various osmiophilic contents, and clusters of free ribosomes (Figs. 3, 4K, 5D, E).

The second and third sheath cells are slender and restricted to the distal half of the sensory apparatus adjoining the dendritic sheath and the whole path of the dendritic outer segments. In cross-sections, both sheath cells first appear below the base of the sensory cone (Figs. 3, 4D, I) and extend down to the area where the dendritic outer segments become coiled (Figs. 3, 4K). The second sheath cell directly encompasses the dendritic sheath only over a short distance of approx. 2–3 µm; precisely where the dendritic sheath thickens (compare Fig. 4I, J). Above that region, the coiled apex of the third sheath cell directly abuts the dendritic sheath up to the sensory cone. Both sheath cells form and surround a small outer sensillum lymph space that displays extremely osmiophilic, most likely liquid contents. This outer sensillum lymph space is traversed by microvilli, which are protruded by both sheath cells (Figs. 3, 4D, I). Towards their periphery, the second and third sheath cell may interdigitate with lateral projections of interstitial regular epidermal cells (Fig. 4D). The cytoplasmic composition widely resembles that observed in the first (scolopale-like) sheath cell, but all organelles, especially mitochondria, are less abundant.

#### Associated exocrine epidermal glands

Towards the periphery of the spacious sensory epithelium, approximately ten solitary recto-canal epidermal glands are present in circular formation (Figs. 4A, 6A). Each gland consists of three different cells (Figs. 3, 6F). The most proximal one, the secretory cell, is bottle-shaped and exhibits a high abundance of rough ER cisternae as well as numerous highly electron-dense secretory granules with an average diameter of 0.5 µm (Figs. 3, 6E). Below the slightly invaginated cell apex (= reservoir), these granules often fuse and adhere to the apical cell membrane. It appears likely that the secretion is discharged by exocytosis (merocrine type of secretion) as the matter visible in the reservoir and the non-cuticularized proximal compartment of the conducting canal show equal electron density (Fig. 6D). The second cell directly overlaying the secretory cell is identified as the intermediary cell (Figs. 3, 6C–F). It is quite small and contains numerous spherical to ovoid, extremely electron-dense granules (0.5–0.7 µm in diameter; Fig. 6C–F). It coils up around the intermediate part of the conducting canal. Its apex is riddled with microvilli invading the conducting canal (Fig. 6C). Only in its most distal part, the apex of the intermediary cell is lined by a cuticle (Fig. 6F). The third cell, the canal cell, occupies about 50% of the entire length of the gland (Fig. 3). Like the intermediary cell, the canal cell is coiled around the distal, always cuticle-lined compartment of the conducting canal, indicated by a mesaxonal membrane (Figs. 3, 6B). The canal cell apex surrounds the distal part of the intermediary cell and is deeply infolded (Fig. 6C). The proximal part of the cell apex contains elongated mitochondria as well as microvilliform processes, which often interlink (Figs. 3, 6C). These processes invade a very large, often electron-lucent extracellular space (intramicrovillar space). It most probably represents a ‘subcuticle’ (layer in between the endocuticle and the epithelium) as it directly abuts the cuticular lining of the conducting canal (Figs. 3, 6B). Distally, the apex of the canal cell forms several microvilli reinforced by microtubules covered by a distinct cuticular layer of the conduction canal (Fig. 6B). Its complex expansion pattern is caused by the strongly infolded apical cell membrane. The tips of microvilliform processes are covered by a highly electron-dense plaque from which extracellular fibers make contact with the inner surface (oriented towards the intermicrovillar space) of the canal cuticle (Fig. 6B). On its passage through the cuticle, the cytoplasm of the canal cell diminishes and finally the conducting canal opens into the cavity of the scape organ (Fig. 3). The secretion forms an extremely electron-dense layer that covers the cavity and, in particular, the surface of the sensory cones.

### Peg-shaped sensilla

**Fig. 7:**
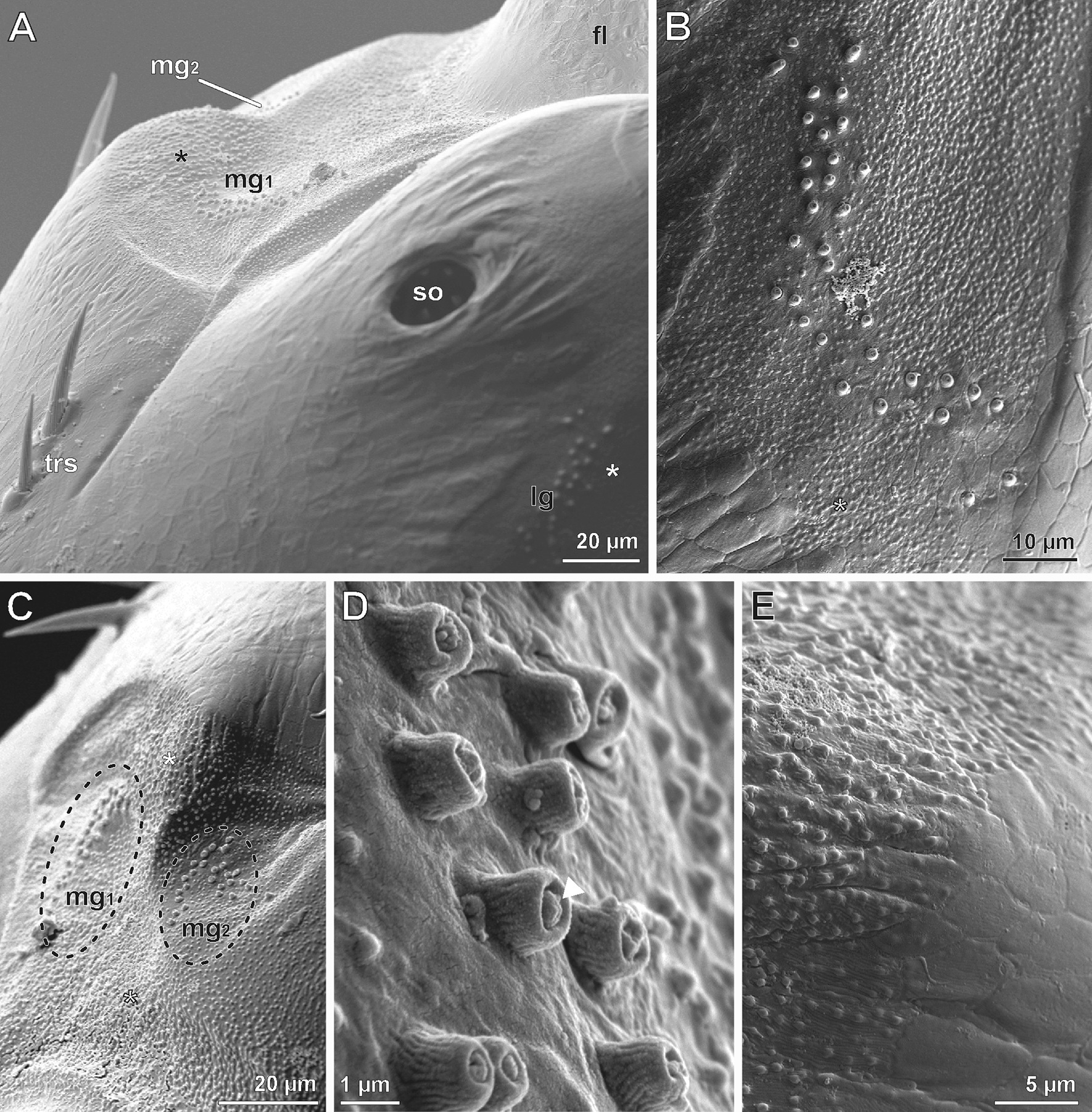
Groups of peg-shaped sensilla outside of the scape organ based on SEM analysis. **A** Second (distal) basal antennomere (right antenna, flagellum to the top right) exhibiting the elevated pore of the scape organ and three groups of peg-shaped sensilla in sickle- or chevron-like formation, situated laterally (lg) and medially (mg_1_, mg_2_). Asterisks mark areas of specialized, tuberculate cuticle framing the groups of peg-shaped sensilla. The two characteristic trichoid sensilla (bottom left) are located medially and line the proximal part of the scape organ. **B** Lateral group of peg-shaped sensilla in detail. Note the surrounding, tuberculate cuticle. **C** Both medial groups of peg-shaped sensilla (indicated by dashed circles) are surrounded by tuberculate cuticle (flagellum to the bottom). Medial group 1 is sickle-shaped and directly abuts the scape organ whereas the pegs in the medial group 2 are arranged like a chevron and are located further away from the scape organ. **D** Detail of peg-shaped sensilla of medial group 1. The pegs only taper slightly at their tip, the circular area is clogged by polymorphic mass (arrowhead). **E:** Transition zone of regular and tuberculate cuticle. On the surface of the regular cuticle, scutes are well visible (compare also Fig. 7B). fl, flagellum; lg, lateral group of peg-shaped sensilla; mg_1_, medial group 1 of peg-shaped sensilla; mg_2_, medial group 2 of peg-shaped sensilla; so, scape organ; trs, trichoid sensillum

**Fig. 8:**
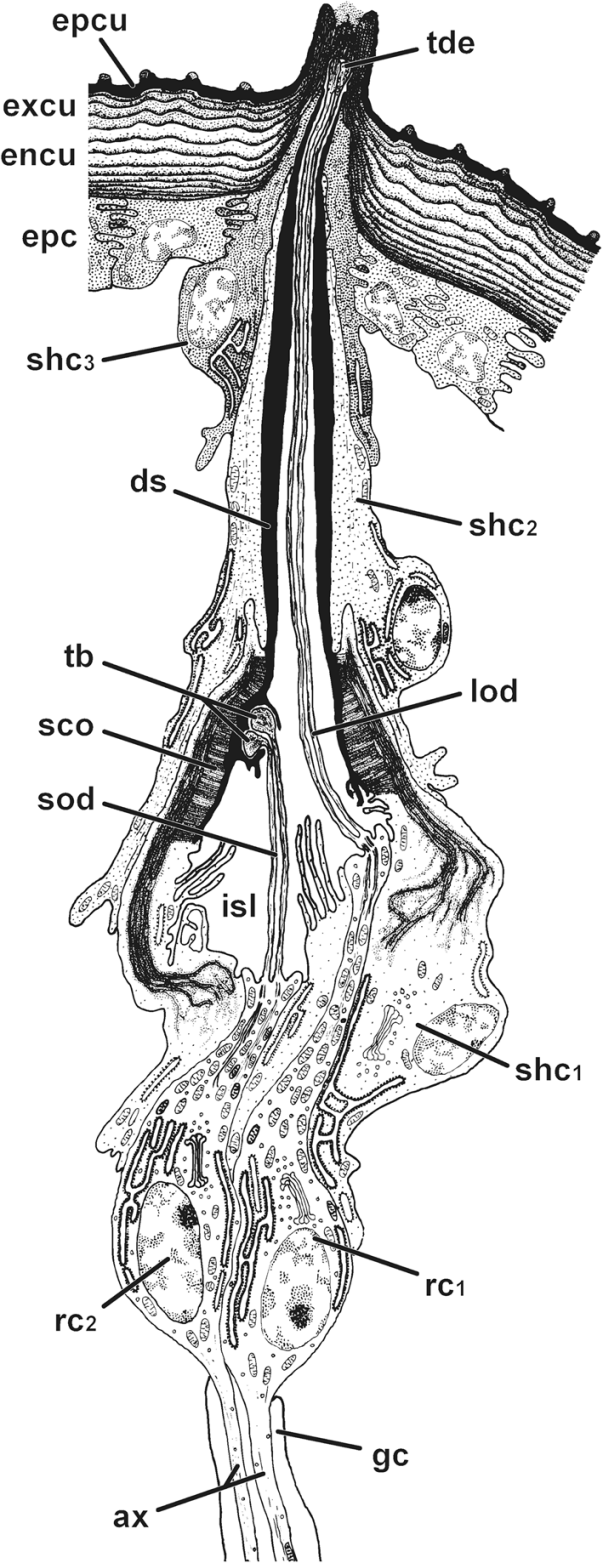
Semi-schematic reconstruction of a peg-shaped sensillum located on the second (distal) antennomere close to the scape organ of *Scutigera coleoptrata* based on TEM analysis. ax, receptor cell axon; ds, dendritic sheath; encu, endocuticle; epc, interstitial epidermal cell; epcu, epicuticle; excu, exocuticle; gc, glial cell; isl, inner sensillum lymph space; lod, long dendritic outer segments; rc_1_, receptor cell with long dendritic outer segments; rc_2_, receptor cell with short dendritic outer segments (scolopidial receptor); sco, scolopale-like structure; sod, short dendritic outer segments; shc_1_, first (proximal) sheath cell (scolopale cell); shc_2_, second (median) sheath cell; shc_3_, third (distal) sheath cell; tb, tubular bodies of short dendritic outer segments; tde, terminal structures of long dendritic outer segments resembling tubular bodies

**Fig. 9:**
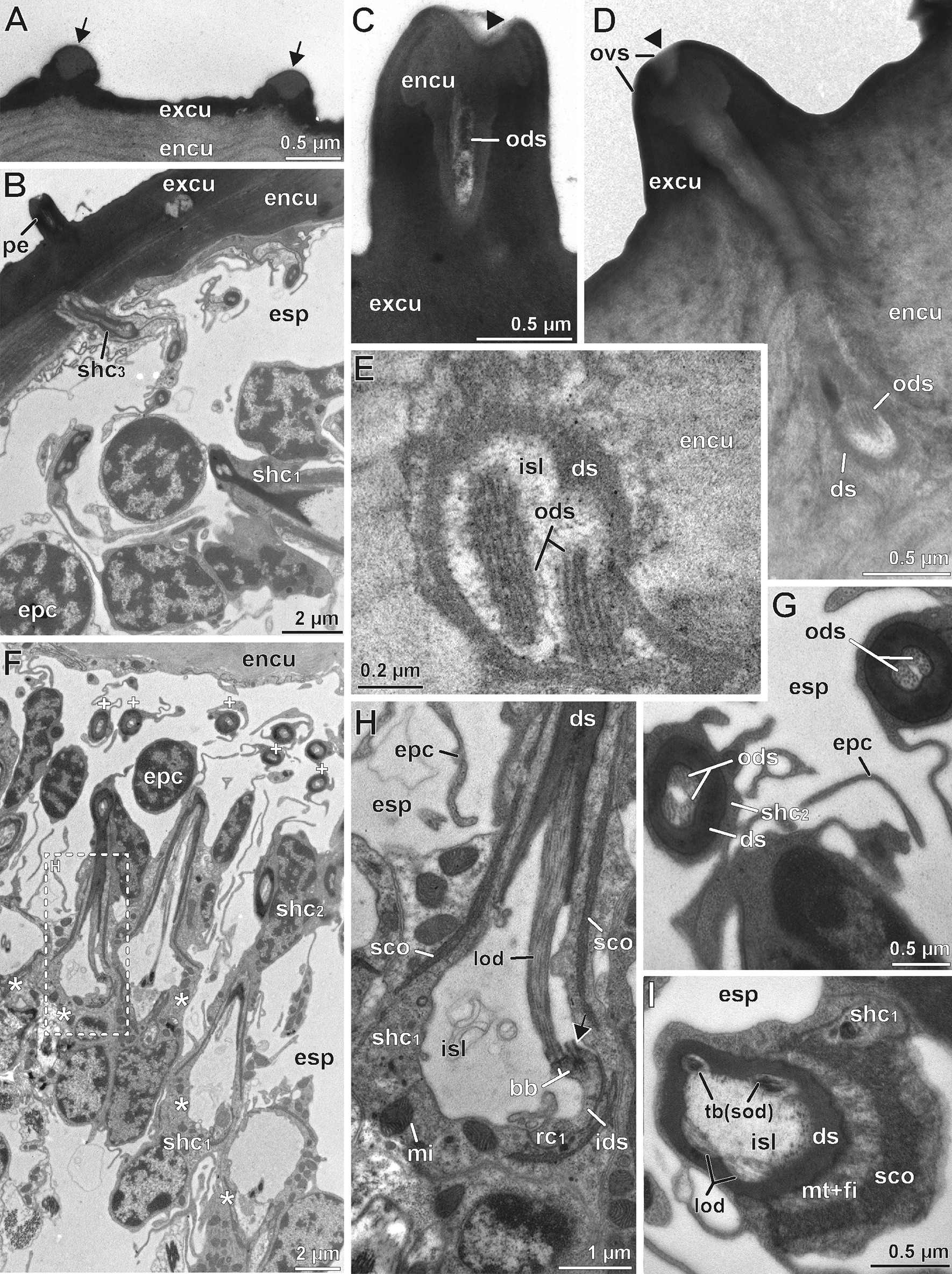
Internal ultrastructure of peg-shaped sensilla based on TEM analysis**. A** Section of the cuticle revealing the peculiar ultrastructure of nano-ridges delimitating the cuticular scutes. Ridges appear rounded due to the presence of conspicuous bars (arrows) lying on and obscuring the groove-like structure established by the much more electron-dense exocuticle. **B** Distal aspect of a peg-shaped sensillum from the lateral group, longitudinal section. Note the coiled nature of the sensillum cell apparatus, which is cut at five different levels (from upper left to lower right). **C** Close-up of the peg cut longitudinally. Only parts of the central mass plugging the tip pore are visible (arrowhead). **D** Longitudinal section of a peg-shaped sensillum showing a long extracellular canal lined by a cuticle. Note that the tip-pore (arrowhead) is clogged by secretion, which is continuous with the layer on the peg shaft. **E** Close-up of profiles of two outer dendritic segments projecting up the extracellular canal. These profiles are suggested to be terminations as they exhibit characteristics of tubular bodies. **F** Longitudinal overview section of several peg-shaped sensilla cut at various levels. The transition zones of inner and outer dendritic segments are surrounded by the first (scolopale) sheath cell (asterisks). Cross profiles of more distally cut peg-shaped sensilla can be found at the upper end of the image (crosses). The inner sensillum lymph space only carries two outer dendritic segments. **G** Distal aspect of peg-shaped sensilla in detail. The two outer dendritic segments of biciliated receptor cell 1 (rc_1_) with elongated dendritic outer segments are enwrapped by a thickened dendritic sheath formed mainly by the second (median) sheath cell. **H** Close-up (dashed box in F) of the transition zone of inner dendritic segments and both long outer dendritic segments of receptor cell 1. One cilium is only represented by its most basal part (arrow). Note the low electron-density of the inner sensillum lymph space contrasting well with the strongly osmiophilic scolopale-like structure in the first (proximal) sheath cell. **I** Transverse-oblique section further proximally of (**G**) showing the region where the two short outer dendritic segments of receptor cell 2 (rc_2_) are attached to the dendritic sheath via tubular bodies. The long outer dendritic segments are cut in the opposite corner of the receptor lymph cavity. Note the extensive system of microtubules and filaments connecting the scolopale-like structure to the outer rim of the dendritic sheath. bb, basal body/bodies; encu, endocuticle; excu, exocuticle; ds, dendritic sheath; epc, interstitial epidermal cell; esp, extracellular space; fi, unspecified cytoplasmic filaments; ids, inner dendritic segment; isl, inner sensillum lymph space; lod, long outer dendritic segments); mi, mitochondrium; mt, microtubules; ods, outer dendritic segment(s); ovs, secretion layer; pe, peg-shaped sensillum; rc_1_, receptor cell 1 with long outer dendritic segments); sco, scolopale-like structure; shc_1,_ first sheath cell (scolopale cell); shc_2_, second (median) sheath cell; shc_3_, third (distal) sheath cell; sod, short dendritic outer segments; tb ,tubular bodies

**Fig. 10:**
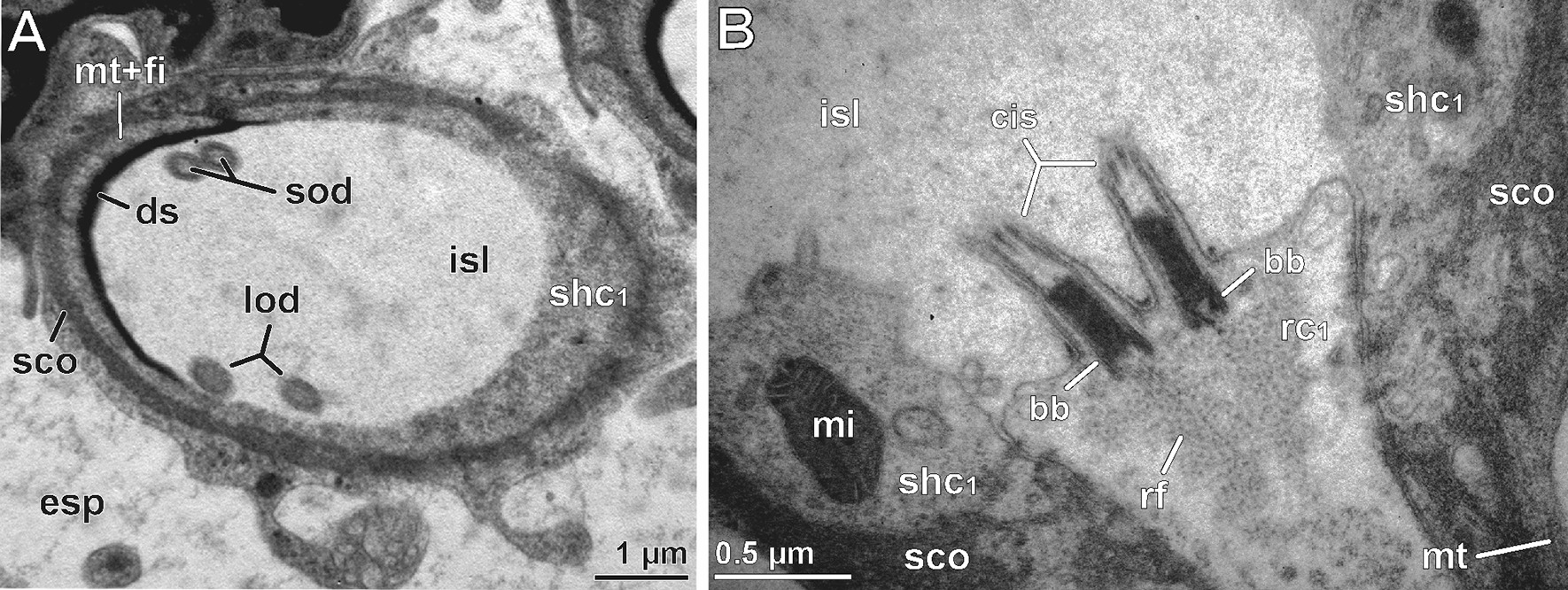
Internal ultrastructure of peg-shaped sensilla based on TEM analysis. **A** Transverse-oblique section of the inner sensillum lymph space and the two pairs of outer dendritic segments, slightly further proximally of the section in Fig. 9I. The scolopale-like structure is still present at one side of the first (proximal) sheath cell. The dendritic sheath is diminished. **B** Longitudinal section of the bottom of the inner sensillum lymph space showing the biciliated receptor cell 1 with both basal bodies and basal parts of long outer dendritic segments, surrounded by the first sheath cell (scolopale cell) with elaborated scolopale-like structure. bb, basal body/bodies; ds, dendritic sheath; esp, extracellular space; fi, unspecified cytoplasmic filaments; isl, inner sensillum lymph space; lod, long outer dendritic segments); mi, mitochondrium; mt, microtubules; rc_1_, receptor cell 1 with long dendritic outer segments); rf, ciliary root filaments; sod, short outer dendritic segments); sco, scolopale-like structure; shc_1_, first sheath cell (scolopale cell)

Three groups of inconspicuous peg-shaped sensilla are present at either side of the bulged elevation of the second antennomere bearing the scape organ (Figs. 2A, 7A). Each group includes a variable number of sensilla, (between 25 and 35) arranged in a sickle- or chevron-like formation (Figs. 2A, 7A–C). Two groups of peg-shaped sensilla are located medially of the scape organ (mg_1_; with approx. 30 sensilla, mg_2_ with approx. 25; Figs. 2A, 7A, C). The third group is located laterad of the scape organ (lg; with approx. 35 sensilla; Figs. 2A, 7A, B). The pegs are about 1 µm long and wide and display a surface pattern of flattened vertical ribs. The tip bears a terminal pore always plugged by a polymorphic mass. In direct vicinity of the pore, the cuticular surface appears smooth (Fig. 7D). A socket is absent and the shaft is firmly affixed to the cuticle (Fig. 7D). Cuticular tubercles of the surrounding exocuticle can be easily identified by their drop-shaped structure (0.2–0.3 µm in length; Figs. 8, 9A). Except for slightly different cell numbers (receptor cells) and few ultrastructural details (e.g. lower electron-density of the inner sensillum lymph space), peg-shaped sensilla widely exhibit the same cellular organization as described for cone-shaped sensilla of the scape organ (compare Figs. 3 and 8). Only two biciliated receptor cells with paired, either long (rc1) or short (rc2) dendritic outer segments are present (Figs. 9I, 10A, B). Long dendritic outer segments (rc1) project through the inner sensillum lymph space lined by a dendritic sheath varying in width (Fig. 9F–H). The dendritic outer segments terminate as tubular bodies at the terminal pore of the apex of the peg. The tubular bodies adjoin a highly electron-dense mass, probably secretion, plugging the terminal pore (Figs. 8, 9C–E). Dendritic outer segments of receptor cell 2 are much shorter and end far below the cuticle in a thickened dendritic sheath where they anchor in a groove-like inner depression (Figs. 8, 9I).

The sheath cell system of peg-shaped sensilla is almost identical to that of cone-shaped sensilla. The first (proximal), second (median), and third (distal) sheath cells are twisted around themselves at their apices, indicated by the presence of a mesaxonal membrane. The first sheath cell tightly encompasses the distal part of the two receptor cell somata as well as the couple of dendritic inner segments along their entire length up to the region of the basal bodies (Figs. 8, 9H, I). As with cone-shaped sensilla, the most characteristic feature of the first (proximal) sheath cell is the complex network of fibers and microtubules agglutinated in an extremely electron-dense mass – a scolopale-like structure (Figs. 8, 9H, I, 10A, B). The second and third sheath cells are flattened and restricted to the distal half of the sensory system, enveloping the dendritic outer segments at the level of the thin dendritic sheath. The second sheath cell directly encompasses the thickened part of the dendritic sheath over a long distance (Fig. 8). Towards its periphery, the third sheath cell sometimes interdigitates with lateral projections of epidermal cells (Fig. 8).

## Discussion

### The scape organ and the cone- and peg-shaped sensilla

The antennal scape organ was first mentioned by Verhoeff [13], but Fuhrmann’s [10] description in *Scutigera coleoptrata* gave a first overview on the external morphology and basic insights into its anatomy (compare Additional file 1A). It was described in a variety of scutigeromorph species: *S. coleoptrata* [10–13], *Seychellonema gerlachi* ([20], their Fig. 7B), *Parascutigera peluda* [18], and in several other species covering all three families (see character matrices in [16, 19, 34]). Thus, the scape organ is a groundplan feature of Scutigeromorpha [15, 35]. In *S. coleoptrata*, Fuhrmann [10] described the oval opening with a dimension of 15–20 µm with a depth of 30 µm, and counted about 20 cone-shaped sensilla inside the cavity. Sombke et al. [11] depicted only two of them in a pseudomaturus (14–16 mm body length, not sexually mature [1]). In this study, we found 7 to 15 cone-shaped sensilla in various adult specimens. As centipedes regularly molt throughout their adulthood [1, 36] these quantitative variations may be correlated with age and, thus, the size of the scape organ. Fuhrmann [10] detected receptor cells contributing to the cone-shaped sensilla (“Sinneszellen”, see Additional file 1D), the distal parts of the outer dendritic segments immediately below the cones (“Terminalstrang der Sinnesorgane”), and the stretched interstitial epidermal cells forming collars around the sensilla. However, there was no report yet on the three fields of peg-shaped sensilla flanking the scape organ. As they are minute and lack typical traits of sensillum shafts, they are hardly detectable (compare Fig. 1B in [11]). The discovery of peg-shaped sensilla at the base of the antennae of *S. coleoptrata* once again indicates that it is necessary to backup scanning electron microscopic data with transmission electron microscopy. As with this case, TEM studies should be considered mandatory not only to support the SEM-based definition of sensillum types but also to fully disclose the structural diversity of arthropod sensilla and epidermal exocrine glands (for latter aspect see also [37]).

Cone- and peg-sensilla are devoid of a socket. The shaft is smooth in cone-shaped sensilla (similar to larger antennal sensory cones, compare [11]) and ribbed in peg-shaped sensilla (similar to small antennal sensory cones, compare [11]). The terminal pores (= tip-pore) of both types are concealed by secretion. The recto-canal epidermal glands surrounding the scape organ secrete an extremely electron dense, presumably hygroscopic layer covering the tips of cone-shaped sensilla. These glands represent a three-cell variant of recto-canal epidermal glands described by Müller et al. [38, 39]. In peg-shaped sensilla, the terminal pores are clogged by a polymorphic substance. Although the origin of this secretion is still uncertain, it seems reasonable that it is produced by epidermal glands present around the sensillar fields at the scape. The fine structural organization only gradually differs in cone- and peg-shaped sensilla. Both exhibit a similar bending of the sensory apparatus (outer dendritic segments, sensillum lymph space, and dendritic sheath) that is surrounded by a voluminous extracellular space (Fig. 9B, F, I). Receptor cells are always biciliated and enwrapped by three sheath cells. The first (proximal) sheath cell includes an elaborated system of microtubules and cytoplasmic fibers embedded in a highly electron-dense structure that resembles a scolopale (Fig. 9H, I), which is more elaborated in cone-shaped sensilla. In both types of sensilla, the short outer dendritic segments of receptor cell 2 (rc2) are connected to the first (proximal) sheath cell with the scolopale-like structure. Differences are the presence of two cells with long outer dendritic segments (rc1) in cone-shaped sensilla *vs.* only one in peg-shaped sensilla (compare Figs. 4H and 9E, H), the extraordinary thickness of the dendritic sheath below the cuticle in peg-shaped sensilla (Figs. 8B, 9G, I), and a much lower electron-density of the inner receptor lymph (compare Figs. 5D, E, and 8F, H). Concerning their function, a detection of shaft deflection can be excluded. Likewise, a chemoreceptive (olfactory or gustatory) function as proposed by Fuhrmann [10] is unlikely. Since receptor cells with long outer dendritic segments (rc1) terminate in structures strongly resembling tubular bodies abutting a plug pin of secretion, primary mechanisms of mechanical receptor stimulation known from thermo- and hygroreceptive sensilla of insects likely come into consideration. According to the spatial coherence of specific ultrastructures and comparisons with similarly organized ciliary systems in sensilla of arthropods others than centipedes, we propose that in cone- and peg-shaped sensilla of *S. coleoptrata* receptor cell(s) with long outer dendritic segments (rc1) most likely function as hygroreceptor(s). Moreover, the receptor cell with short outer dendritic segments (rc2), along with its interconnection to a scolopale-like structure in the first (proximal) sheath cell, represents a component found in scolopidia of crustaceans and insects, potentially suited for vibration or strain detection.

### Hygroreceptive component—receptor cells with long dendritic outer segments (rc1)

The possibility that receptor cells with long outer dendritic segments (rc1) are involved in hygroreception is based on three structural characters identified by Yokohari [30, 40] in *Periplaneta americana*. (1) They are associated with short, cone- or peg-shaped cuticular shafts with inflexible sockets and no wall pores (a tip-pore may occur) that are similar to hygroreceptors of some hexapods and arachnids (e.g. ‘no-pore sensilla’ in Lepidoptera and Coleoptera [41] and ‘tip-pore sensilla’ in *Cupiennius salei* (Trechaleidae) [42]). (2) They possess paired, but unbranched outer dendritic segments. (3) Tubular bodies are present at the distal ends of the outer dendritic segments connecting to the inner face of the tip cuticle (see summary in [29]). Functional similarities to cone- and peg-shaped sensilla in *S. coleoptrata* concern the mechanisms of both mechano-electric transduction (mechanical deformation of the dendritic tip) and impulse generation (induction at the level of receptor cell somata). In hexapods, the tip cuticle (without a terminal pore, hence ‘np-sensilla’) serves as a connector for the tubular bodies, as has been observed in the sensillum capitulum of *P. americana* [30, 43–45], in the sensillum coelocapitulum of *Apis mellifera* [46], and in the sensilla coeloconica of *Locusta migratoria* [47] or *Carausius morosus* [31]. In blunt-tipped peg sensilla on larval antennae of *Tenebrio molitor*, two tubular bodies are nested in a terminal network of the dendritic sheath, which is subjacent to a central canal connected to a tip-pore [48]. Similar to *T. molitor* and *C. salei*, in cone- and peg-shaped sensilla of *S. coleoptrata* there is a terminal tip-pore, but it is completely or partly plugged by an electron-dense substance, which most likely represents secretion released by recto-canal epidermal glands (compare [38]). This secretion may be hygroscopic and expand if the animal enters moist areas. It is likely that, if moistened, the secretion plug may elongate and distort the subjacent tubular bodies, thus transducing a mechanical stimulus on these receptor cells. Thus, the hygroscopic material, which is most likely produced by the associated epidermal glands, may drive this hygro-mechano transformation. The deformation of the tubular body-like structures, as it is here assumed to be achieved by the swelling of overlaying secretion, distinctly differs from hexapod hygroreceptors where the transformation is achieved by deformation of the cuticle [29].

The possible triggering of hygroreceptors utilizing secretion plug systems seems to be a new finding in arthropod sensilla research. Regrettably, the literature is ambiguous with respect to the spatial coherence and functional role of so-called molting pores in hexapod hygroreceptive sensilla. In their review on invertebrate hygroreceptors, Altner and Prillinger [23] did not regard the molting pore as crucial for stimulus transmission. Reassessing drawings and TEM micrographs provided by other authors, led us to the conclusion that the molting pore is plugged by an electron-dense material that makes connection to the inner sensillum lymph space [27, 29, 31, 41, 49]. Biochemical properties aside, this system of hygroscopic material being in close contact to the tubular bodies, as revealed in Hexapoda, may be generally comparable to that in *S. coleoptrata*. In hexapods, swelling or shrinking of hygroscopic sensillum structures result in a deformation/distortion of and voltage changes across the tubular bodies of (type-1) receptor cells (compare [41, 50] and citations therein). In the hexapod model, the hygroscopic transducer is the cuticle, in *S. coleoptrata* it is secretion. Both are a functional morphological prerequisite of hygroreceptors operating according to the mechanical hygrometer model as defined by Tichy and Loftus [29]. Thus, we propose to add secretion plugging the tip-pore as a potential transducer. In the hygroreceptive tip-pore sensilla of the wandering spider *Cupiennius salei*, the terminal pore is not plugged, but humidity-induced changes are recorded by electrolyte concentrations in the receptor lymph (electrochemical hygrometer model) [29, 42, 51].

The presence of biciliated hygroreceptor cells (as in cone- and peg-shaped sensilla in *S. coleoptrata*) is uncommon in hexapods (so far only found in Collembola [28]), but has been found in some myriapod species associated with mechano- and probably hygroreception [5, 32]. Possessing two cilia instead of a single one may result in an increased sensitivity of a given hygroreceptor cell (compare [30]). However, a combination of hygroreceptive with thermoreceptive elements, present in many hexapods as ‘triads’ [23, 27, 29, 30, 52, 53], is absent in *S. coleoptrata*. In myriapods, hygroreception is not restricted to the antennal base. In another scutigeromorph species, *Thereuonema tuberculata*, humidity and mainly CO_2_ reception was electrophysiologically recorded as a function of the organ of Tömösváry (= postantennal organ), which is located between the compound eye and the antennal base on both sides of the head [54, 55]. There, dendritic outer segments of biciliated receptor cells branch distally and terminate close to a thin cuticle; tubular bodies are absent [55]. A similar organization of postantennal organs (also in association with epidermal glands) was described for *Lithobius forficatus* [56, 57] and for millipedes and symphylans [58, 59]. Here, receptor cells are likewise biciliated, but stimulus transduction is different from the one proposed for cone- and peg-shaped sensilla in *S. coleoptrata*.

### Scolopidial component—receptor cells with short dendritic outer segments (rc2)

Typical features are a single biciliated receptor cell with short dendritic outer segments transforming distally into tubular bodies. They nest in cavities of the proximal region of the dendritic sheath that is connected to a scolopale-like structure in the first (proximal) sheath cell. The presence of tubular bodies indicates a mechanical pathway of stimulus transduction while the scolopale-like structure reveals close structural and functional resemblance with sensilla containing scolopidial elements. In Arthropoda, there are internal and external mechanoreceptors (see reviews [26, 60–62]). In terrestrial arthropods, external mechanoreceptors are either uni- or bimodal hair-like (trichoid) sensilla (including campaniform sensilla). The dendritic outer segments of their receptor cells attach to the proximal end of the sensillum shaft via a typical tubular body [26]. In crustaceans, external mechanoreceptors are exclusively equipped with a scolopale [63–65]. Internal mechanoreceptors described in Pancrustacea have been assigned to the class of scolopidia (reviews [26, 62, 66–68]). If aggregated, the scolopidia act as functional units in multimodular mechanoreceptive sense organs, usually termed chordotonal organs [62]. Hexapod and crustacean scolopidia as well as crustacean mechanoreceptive (scolopidial) hair sensilla are characterized by: (1) one to four bipolar (type-1) receptor cells, (2) one or several attachment cell(s) at the distal end, (3) one or several proximal glial cell(s) surrounding the proximal region of receptor cells, and (4) a peculiar structure, called the scolopale, which is located within the innermost sheath cell, called the scolopale cell [61–63, 67–69]. The scolopale consists of an electron-dense matrix of aggregated proteins traversed by actin filaments and longitudinally arranged microtubules. In hexapod scolopidia, the scolopale may extend and diverge into a cylindrical complex of distinct scolopale rods strengthening distal finger-like processes of the scolopale cell (e.g. [70]). The scolopale cell surrounds the inner sensillum lymph space and the inner dendritic segments of the receptor cells, which house prominent ciliary rootlets in their dendritic inner segments [26, 67]. The attachment cell(s) of hexapod and crustacean scolopidia encompass proximally an extracellular structure, the so-called (scolopale) cap or tube [67, 69]. The typology of hexapod and crustacean scolopidia is rather complex and refers to various ultrastructural features. These include proportions of the dendritic outer segments, without (type-1) or with (type-2) distal dilation. Additionally, it refers to number and appearance of receptor cells, or the extracellular structure associated with dendritic outer segment(s) and the scolopale cell. In the latter, dendritic tips can be firmly inserted in a scolopale cap (mononematic) or just surrounded by an electron-dense tube (amphinematic)) [69]. The scolopidial component of cone- and peg-shaped sensilla of *S. coleoptrata* shares some ultrastructural traits with mononematic, type-1 scolopidia: (1) the position of the scolopale cell (first sheath cell surrounding the ciliary region), (2) the scolopale-like structure that at least in part shows a rod-like packing, (3) short dendritic outer segments with a slightly swollen terminal structure that (4) are firmly anchored into an extracellular structure that is the dendritic sheath.

Interestingly, in hexapods and crustaceans, the scolopale cap has never been consistently termed as a dendritic sheath, but should be identified as such because it is not in contact with the surface cuticle, it lines the inner sensillum lymph space, and is most likely secreted by the scolopale cell (e.g. [67, 71, 72]). Following the typical sequence of sheath cells in cuticular sensilla of arthropods, the attachment cell would be homologous to the second (trichogen) sheath cell as it overlays the scolopale cap and contains microtubules in high abundances (e.g. [67, 69, 73]). However, some ultrastructural details such as local dilations in the dendritic outer segment(s) below the cap level, or apical processes of the scolopale cells containing scolopale rods and piercing the attachment cell, are unique characters of crustacean and hexapod scolopidia (e.g. [67, 69]). The presence of two cilia as well as the presence of axial/perpendicular microtubules are two unique features of the receptor cells in cone- and peg-shaped sensilla of *S. coleoptrata*.

### Affinities of cone- and peg-shaped sensilla among arthropods

The tip-pore sensilla (antennal cone- and peg-shaped sensilla associated with the scape organ of *Scutigera coleoptrata*) described here represent a new type of bifunctional arthropod sensilla that combine hygroreceptive and scolopidial components associated with long (rc1) and short (rc2) dendritic outer segments. Similar cone- and peg-shaped sensilla in *S. coleoptrata* occur in moderate numbers on the antennal nodes, which were interpreted as proprioceptors because of their specific location [11]. Peg-shaped sensilla are also present at the base of locomotory legs in *S. coleoptrata* (Sombke et al. pers. obs.), which makes it likely that similar sensory structures in general could be involved in possible hygro- and pressure-reception at the base of appendages. It is unclear whether secretions also act as general transducers, but the epidermis of centipedes is generally rich in solitary epidermal glands [1, 37–39, 74]. Hence, the clogging of terminal pores of peg-shaped sensilla would be potentially provided by those glands.

Similar arthropod sensilla equipped with short cones, inflexible sockets and scolopale-like structures in the first sheath cell have been recorded only in combined hygro- and thermoreceptors of some hexapods that are summarized as ‘no-pore sensilla with inflexible socket’ [27, 30, 73, 75, 76]. They include two types of receptor cells: type-1 receptor cells with long dendritic outer segments (referred to as hygroreceptors) and type-2 receptor cells with short dendritic outer segments. The dendritic tips of the type-2 receptor cells often exhibit a strongly infolded or lamellated apical membrane, a pattern which is considered suitable for thermoreception (e.g. [28, 31]). However, lamellated dendritic tips are absent in cone- and peg-shaped sensilla of *S. coleoptrata*. The cytoskeletal elements of the scolopale equivalent in hexapod *np*-sensilla are thought to stabilize the inner (thecogen) sheath cell, which consequently protects the outer dendritic segments against mechanical forces [47, 73]. Frequently, a type-3 receptor cell is present with a short and thin dendritic outer segment that exhibits an undifferentiated cilium [28]. In the larval blunt-tipped peg sensillum of *Tenebrio molitor* this cilium terminates near a scolopale-like structure close to the dendritic sheath [70], which is strikingly similar to the receptor cell with short cilia (rc2) in cone- and peg-shaped sensilla of *S. coleoptrata.* In addition, blunt-tipped peg sensilla in *T. molitor* bear a terminal (molting) pore that is distinctly constricted and plugged with an electron-dense material [70]. Thus, these sensilla can be considered functional equivalents (except for thermoreception) to cone- and peg-shaped sensilla in *S. coleoptrata*. Admittedly, as hygroreceptive sensilla must have evolved after the independent conquest of land by myriapods and hexapods [77, 78], these systems are very likely convergent transformations of an already exsiting sensillar type. Scolopidia (sensilla with scolopidial elements) are discussed as a possible ancestral type of sensilla in Pancrustacea (e.g. [63, 68, 79, 80]). However, it is not clear whether scolopidia of crustaceans and hexapods evolved independently or may share a common evolutionary origin with bifunctional sensilla containing scolopodial components as described here for *S. coleoptrata*. At least mononematic scolopidia may arguably represent an ancestral mechanoreceptor system in Mandibulata that contained externally visible sensilla with scolopidial components. As outlined above, the scolopale cap may be homologized with the dendritic sheath and would be the result of transformation and compression. Within myriapods, penicillate millipedes (*Polyxenus lagurus*) possess bifunctional hygro- and thermoreceptive np-sensilla, termed sensilla coeloconicum and setiform sensillum S’ [32, 81, 82]. However, both types differ from the cone- and peg-shaped sensilla of *S. coleoptrata* by the presence of lamellated dendritic outer segments (indicative of thermoreception). Nevertheless, both types of sensilla possess similar biciliated receptor cells with short outer dendritic segments. The short outer dendritic segments in the sensillum coeloconicum of *P. lagurus* lack tubular bodies and get in close contact with the dendritic sheath near the socket of the sensilla. However, in the setiform sensillum S’, the tips of two cilia of one receptor cell terminate in tubular bodies tightly encompassed by the dendritic sheath and apical processes of the inner sheath cell. There, apical processes are strengthened by microtubules and were therefore termed as ‘ciliary-like structures’ [32, 81]. These cytoskeletal structures are probably equivalent to the ‘scolopale rod-like structures’ described in the sensillum capitulum of *Periplaneta americana* [43]. Rilling [83, 84] mentioned scolopidia-like sense organs in the mandible and maxilla of the centipede *Lithobius forficatus*, but based on drawings of methylene blue stained sections, a clear identification as scolopidia is not possible. Nonetheless, the presence of scolopidia-like components was described before (at least) in Diplopoda. Consequently, we propose them to belong to the groundplan of Myriapoda if not even of Mandibulata. The latter assumption would stand in line with reassessments of other anatomical characters, which were previously thought to be present in Pancrustacea alone [85–88].

## Methods

### Field collection and dissection

Specimens of *Scutigera coleoptrata* (Linnaeus, 1758) (Fig. 1A) were collected from interstices of rock piles and/or rotting tree trunks located in pine tree forests from three different sampling sites: (1) Cala Llenya (Ibiza, Balearic Islands, Spain), (2) La Couronne (approx. 20 km west of Marseille, France), and (3) Pomer (Pula, Istrian Peninsula, Croatia) in 2012. Twelve adult individuals were investigated using various microscopic methods. Specimens were anaesthetized with CO_2_ for at least 10 min. Prior to fixation, two individuals were examined using a Keyence VHX digital microscope to obtain images from the scape organ in live animals.

### Scanning electron microscopy (SEM)

The heads of five specimens were fixed overnight in Bouin’s solution at room temperature (e.g. [89, 90]). After dehydration in a graded series of ethanol, heads were critical point dried (Leica EM CPD300), mounted on coiled copper tape, coated with silver conductive adhesive, sputter-coated with gold, and examined with a ZEISS EVO LS10 (Imaging Facility of the Department of Biology, University of Greifswald).

### Transmission electron microscopy (TEM)

Pieces of the basal antennal region of four specimens were incubated in a modified fixative solution after Karnovsky [91] containing 2.5% glutaraldehyde, 2.5% paraformaldehyde, 1.5% NaOH and 1,5% D-glucose (in 0.1 M sodium phosphate buffer, pH 7.4) at room temperature. For methodological comparison, Karnovsky-based fixation of antennal pieces of one specimen was conducted in a Biowave 3450 (TedPella) by applying 3 × 2 min pulses. After rinsing three times for 5 min in buffer solution, post-fixation in 2% OsO_4_ solution was conducted at room temperature for 3 h, followed by dehydration in graded series of ethanol and embedding in Araldite (Fluka), EponEmBed 812, or Spurr media (Sigma Aldrich). Ultrathin sections (55–70 nm) were prepared using a Leica UCT ultramicrotome. Serial ultrathin sections were mounted on Formvar-coated slot grids (PLANO, G2500C), stained with uranyl acetate and lead citrate for 4 min, and examined under a ZEISS 902 (Institute of Pathology, University Hospital Essen, Germany) and a JEOL JEM-1011 (General and Systematic Zoology, University of Greifswald, Germany) transmission electron microscope operated at 80 kV. Digital micrographs were obtained with the aid of mid-mount cameras (Morada: ZEISS 902, Essen; Megaview III, Soft Imaging System, Greifswald) using iTEM imaging software.

### Histology

The dissected heads were fixed in Bouin’s solution, dehydrated through a graded series of ethanol, terpineol and xylenes, and finally embedded in paraffin (Kendall Paraplast Plus, Tissue Embedding Medium, Tyco Healthcare). Sections (10 µm) through the scape organ were prepared with a Microm HM-360 rotary microtome, mounted on coated glass slides and silver-stained after a modified protocol based on Bodian [92]. Staining procedure included (1) rehydration through xylenes, terpineol, and a graded series of ethanol, (2) incubation in solution of 250 ml H_2_O and 2.5 g silver protein (protargol) with addition of 6 g cleaned copper pellets for 24 h at 60 °C, (3) brief washing in H_2_O, (4) developing in a solution containing 200 ml H_2_O, 2 g hydroquinone, and 4 g sodium sulfite, (5) washing in a gentle current of tap water for 3 min, (6) intensifying in 1% gold chloride for 10 min under strong artificial light, (7) washing in two changes of H_2_O (30 s), (8) reducing in 200 ml H_2_O with 4 g oxalic acid (8 min), (9) washing in two changes of H_2_O (30 s), (10) removing residual silver salts in 200 ml H_2_O with 10 g sodium thiosulfate (5 min), (11) washing in two changes of H_2_O (8 min), (12) dehydration through a graded series of ethanol, terpineol, and two changes of xylenes, and (13) immediate embedding using Entellan (Electron Microscopy Sciences) or Roti-Histokitt II (Carl Roth) mounting media. Additionally, one specimen was fixed in Bouin’s solution, washed in several changes of phosphate buffered saline, dehydrated in a graded ethanol series, incubated in a 1:1 solution of ethanol and tetrahydrofuran (Carl Roth, CP82.1) for 2 h, pure tetrahydrofuran for 18 h, and in a solution of 1:1 tetrahydrofuran and paraffin (Carl Roth, 6643.1) at 60 °C for 24 h. Finally, samples were embedded in pure paraffin and sectioned (5 μm) with a motorized rotary microtome (Microm HM 360). Sections were stained with Azan according to Geidies [93] and mounted in Roti-Histokitt II (Carl Roth, T160.1). Light micrographs were taken with an Olympus BX60 microscope equipped with an AxioCamMRc digital camera and a Nikon Eclipse 90i microscope.

### Terminology

Terminology in the field of arthropod sensilla is diverse and often not consistently applied among various taxa. Their description and typology are often based only on external characters (e.g. by SEM analysis). In addition, in the literature, the terms ‘modality’ and ‘function’ are often used as synonyms (e.g. [27]). In the present study, we generally distinguish between receptor modalities of mechanoreception and chemoreception (electroreception is the third possible modality). On the functional level, we can even specify more types e.g., pure mechanoreception, gustation, olfaction, thermoreception, hygroreception, phonoreception etc. Thus, a contact-chemoreceptive sensillum is bimodal and bifunctional; a thermo/hygroreceptive sensillum is unimodal, but bifunctional (as both transductions are realized via mechanoreceptive elements; e.g. [48]).

## Supplementary Information

**Additional file 1**. Previous morphological survey of the basal antennal region and the scape organ of *Scutigera coleoptrata* modified after Fuhrmann [10]. Wherever possible, Fuhrmann’s original labels were synonymized and replaced. Original German terms are given in brackets in the list of abbreviations below. **A** Basal antennal region. **B** Dorsal aspect of the scape organ. **C **Cross-section of the second (distal) antennomere. **D **Medio-longitudinal section of the scape organ in higher detail. aa, antennal artery (=*art *Arterie); am, ampulla (= *amp *Ampulle); amu, antennal muscle fibers (= *mu/mu*_*2*_ Muskelfasern); anv, antennal nerve (= *Ne *Hauptnervenstamm); br, bristles, not further identified (= *b*_*2*_ Typ 2 Borsten); ca, cavity (= *dupl*. Hautduplikatur); co, sensory cones (= *z *Sinneszapfen); de+dos, dendritic sheath and dendritic apparatus (= *term.str. *Terminalstrang); ecm, extracellular matrix (=*bas.membr. *Basalmembran); epc, epidermal cell (= *epze *Epidermiszelle); exc, exo- and epicuticle (= *gr.h. *Grenzhäutchen); gp, gland pore (= *drp *Drüsenpore); hec, hemocytes (= *blut.ze *Blutzellen); hi, hinge/soft intersegmental part of the cuticle (= *gel *Gelenk); nb, neurite bundle (=*ne*_*2*_ Nervenstamm); nc, neuronal cells (= *s.ze *Sinneszellen); nl, neurilem (= *ne.sch *Nervenscheide); r_1/2_, ridge-shaped dorsal protuberances of second (distal) antennomere (= *r*_*1/2*_ Zapfen 1/2); rc, receptor cell (= *sze*Sinneszelle); sc, secretory cell (=*dr.ze *Drüsenzelle); scu, specialized cuticle (=*gel.h *Gelenkhäutchen); so, scape organ (=*shaft.org *Schaftorgan); str, sensilla trichodea (=*b*_*1*_ Typ 1 Borsten); tri, trichomes (= *haut.haar *Häutungshaare); 1, first (proximal) basal antennomere (= *Schaftglied*).

## Data Availability

The data generated and/or analyzed during the current study are available from the corresponding authors upon reasonable request.
