# Supplementary figures and images for: The antennal scape organ of Scutigera coleoptrata (Myriapoda) and a new type of arthropod tip-pore sensilla integrating scolopidial components
Source: Front Zool. 2021 Nov 4;18:57. doi: 10.1186/s12983-021-00442-9 (PMC8567564; doi:10.1186/s12983-021-00442-9)

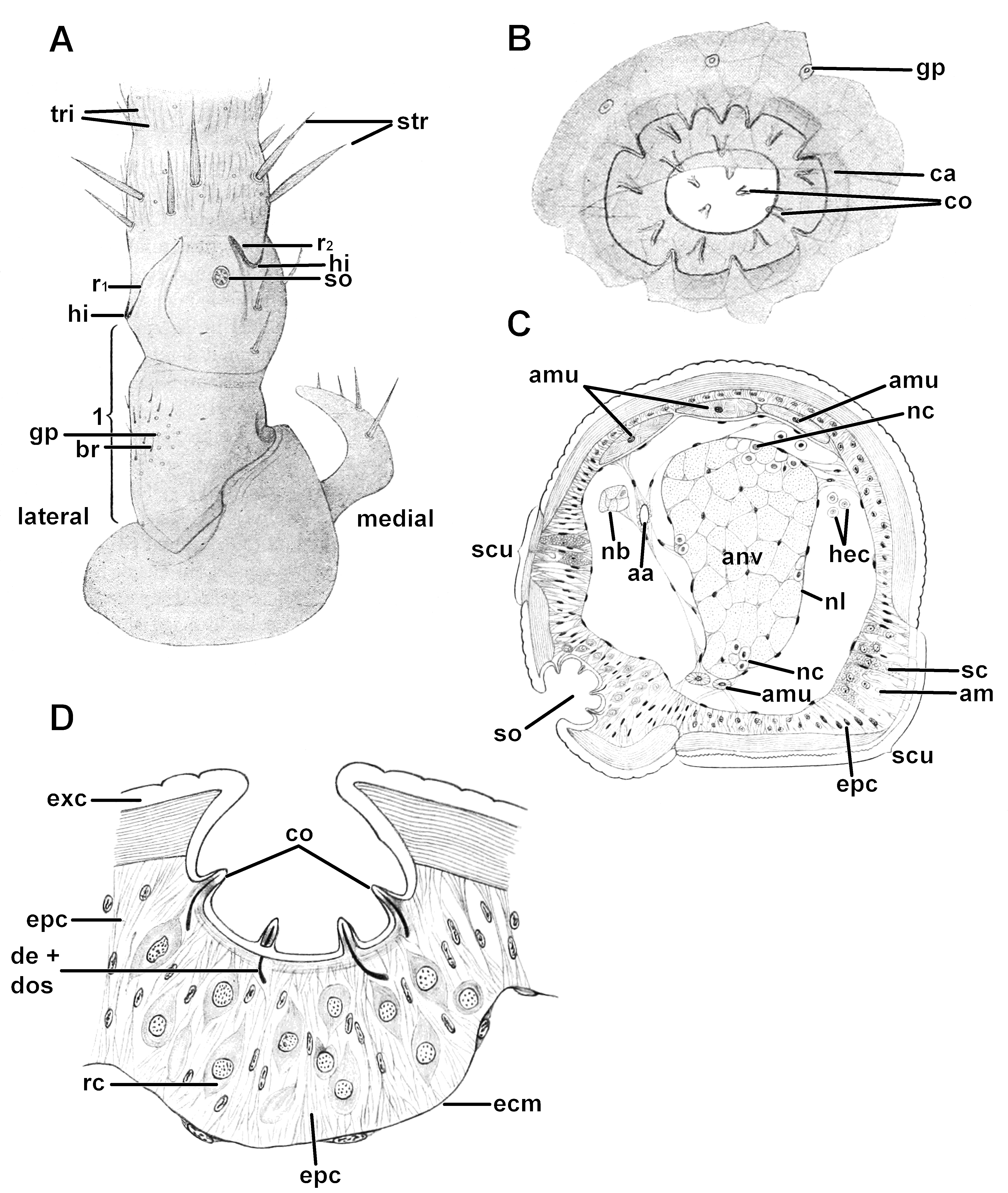

Supplement: Supplementary file 1 — Additional file 1. Previous morphological survey of the basal antennal region and the scape organ of Scutigera coleoptrata modified after Fuhrmann [10]. Wherever possible, Fuhrmann’s original labels were synonymized and replaced. Original German terms are given in brackets in the list of abbreviations below. A Basal antennal region. B Dorsal aspect of the scape organ. C Cross-section of the second (distal) antennomere. D Medio-longitudinal section of the scape organ in higher detail. aa, antennal artery (=art Arterie); am, ampulla (= amp Ampulle); amu, antennal muscle fibers (= mu/mu2 Muskelfasern); anv, antennal nerve (= Ne Hauptnervenstamm); br, bristles, not further identified (= b2 Typ 2 Borsten); ca, cavity (= dupl. Hautduplikatur); co, sensory cones (= z Sinneszapfen); de+dos, dendritic sheath and dendritic apparatus (= term.str. Terminalstrang); ecm, extracellular matrix (=bas.membr. Basalmembran); epc, epidermal cell (= epze Epidermiszelle); exc, exo- and epicuticle (= gr.h. Grenzhäutchen); gp, gland pore (= drp Drüsenpore); hec, hemocytes (= blut.ze Blutzellen); hi, hinge/soft intersegmental part of the cuticle (= gel Gelenk); nb, neurite bundle (=ne2 Nervenstamm); nc, neuronal cells (= s.ze Sinneszellen); nl, neurilem (= ne.sch Nervenscheide); r1/2, ridge-shaped dorsal protuberances of second (distal) antennomere (= r1/2 Zapfen 1/2); rc, receptor cell (= szeSinneszelle); sc, secretory cell (=dr.ze Drüsenzelle); scu, specialized cuticle (=gel.h Gelenkhäutchen); so, scape organ (=shaft.org Schaftorgan); str, sensilla trichodea (=b1 Typ 1 Borsten); tri, trichomes (= haut.haar Häutungshaare); 1, first (proximal) basal antennomere (= Schaftglied). [file 12983_2021_442_MOESM1_ESM.tif]
